# Regulation of xylose metabolism in recombinant *Saccharomyces cerevisiae*

**DOI:** 10.1186/1475-2859-7-18

**Published:** 2008-06-04

**Authors:** Laura Salusjärvi, Matti Kankainen, Rabah Soliymani, Juha-Pekka Pitkänen, Merja Penttilä, Laura Ruohonen

**Affiliations:** 1 VTT, Technical Research Centre of Finland, P.O. Box 1000, FI-02044 VTT, Finland; 2 Structural Genomics, Institute of Biotechnology, P.O. Box 56, University of Helsinki, FI-00014 HY, Finland; 3 Protein Chemistry Unit, Institute of Biomedicine, Anatomy Biomedicum-Helsinki, P.O. Box 63, University of Helsinki, FI-00014 HY, Finland

## Abstract

**Background:**

Considerable interest in the bioconversion of lignocellulosic biomass into ethanol has led to metabolic engineering of *Saccharomyces cerevisiae *for fermentation of xylose. In the present study, the transcriptome and proteome of recombinant, xylose-utilising *S. cerevisiae *grown in aerobic batch cultures on xylose were compared with those of glucose-grown cells both in glucose repressed and derepressed states. The aim was to study at the genome-wide level how signalling and carbon catabolite repression differ in cells grown on either glucose or xylose. The more detailed knowledge whether xylose is sensed as a fermentable carbon source, capable of catabolite repression like glucose, or is rather recognised as a non-fermentable carbon source is important for further engineering this yeast for more efficient anaerobic fermentation of xylose.

**Results:**

Genes encoding respiratory proteins, proteins of the tricarboxylic acid and glyoxylate cycles, and gluconeogenesis were only partially repressed by xylose, similar to the genes encoding their transcriptional regulators *HAP4*, *CAT8 *and *SIP1-2 *and *4*. Several genes that are repressed via the Snf1p/Mig1p-pathway during growth on glucose had higher expression in the cells grown on xylose than in the glucose repressed cells but lower than in the glucose derepressed cells. The observed expression profiles of the transcription repressor *RGT1 *and its target genes *HXT2-3*, encoding hexose transporters suggested that extracellular xylose was sensed by the glucose sensors Rgt2p and Snf3p. Proteome analyses revealed distinct patterns in phosphorylation of hexokinase 2, glucokinase and enolase isoenzymes in the xylose- and glucose-grown cells.

**Conclusion:**

The results indicate that the metabolism of yeast growing on xylose corresponds neither to that of fully glucose repressed cells nor that of derepressed cells. This may be one of the major reasons for the suboptimal fermentation of xylose by recombinant *S. cerevisiae *strains. Phosphorylation of different isoforms of glycolytic enzymes suggests that regulation of glycolysis also occurred at a post-translational level, supporting prior findings.

## Introduction

On hexose sugars, the yeast *Saccharomyces cerevisiae *has an exceptional capacity for rapid anaerobic growth and fermentation of sugar to carbon dioxide and ethanol. However, *S. cerevisiae *exhibits only negligible metabolism of xylose even under aerobic conditions [1,2]. The interest in production of fuel ethanol from renewable plant material, often rich in pentose sugars such as xylose, has encouraged extensive metabolic engineering of *S. cerevisiae *for xylose metabolism [3,4].

Recombinant xylose-utilising *S. cerevisiae *strains have been constructed by introducing xylose reductase (XR; preferring NADPH over NADH) and xylitol dehydrogenase (XDH; strictly NAD^+ ^specific) encoding genes from the xylose-fermenting yeast *Pichia stipitis*, and additionally overexpressing the endogenous xylulokinase encoding gene (*XKS1*) [5-8]. This oxidoreductive xylose pathway has enabled xylose fermentation by *S. cerevisiae *[7,8] but with significantly lower rates compared with glucose fermentation. The different cofactor specificities of XR and XDH disturb the cellular redox cofactor balance during xylose metabolism, particularly under anaerobic conditions. Recently, an evolutionarily engineered strain with xylose isomerase from the anaerobic fungus *Piromyces *sp. E2 was shown to have improved anaerobic growth and fermentation on xylose compared with *S. cerevisiae *strains expressing the oxidoreductive pathway [9-11]. However, even this strain utilised glucose and xylose sequentially, with preference for glucose, and the anaerobic specific growth rate remained lower on xylose than on glucose [11]. It thus seems that the rate of xylose metabolism in engineered *S. cerevisiae *is not solely restricted by problems in the balance of redox cofactors. Uptake of xylose [12] and the low capacity of the pentose phosphate pathway (PPP) in *S. cerevisiae *have been identified as plausible limitations of xylose metabolism in this yeast [5,13].

The overall metabolism and changes generated by mutagenesis in xylose-metabolising recombinant *S. cerevisiae *strains have been analysed by transcriptional, proteome and metabolic flux analyses [14-20]. A moderate increase in transcript levels of some of the genes encoding enzymes of the PPP, as well as in the flux through the pathway was observed in cells metabolising xylose [14,16,18]. Additionally, expression of several genes encoding redox-related enzymes was enhanced [18,21]. Starvation-related but not general stress-related responses were activated during growth on xylose, and furthermore, metabolism on xylose seemed neither fully fermentative nor fully respiratory [21]. Importantly, anaerobic growth on xylose was suggested to be limited by the rate of ATP production [18]. Jin *et al*. compared the transcription of xylose-metabolising *S. cerevisiae *growing on xylose alone or on glucose alone in shake flask batch cultures under aerobic and oxygen-limited conditions [17]. They observed up-regulation of genes encoding activities of the tricarboxylic acid (TCA) cycle and gluconeogenesis, and of respiratory genes under oxygen limitation in cells grown on xylose compared with glucose repressed cells.

Most studies of xylose metabolism in the recombinant *S. cerevisiae *have focused on assessing the reasons behind its inability to grow on or to ferment efficiently this pentose sugar under anaerobic conditions. However, the effect of xylose on the dominant regulatory circuit of carbon metabolism, *i.e*. glucose repression, has so far been largely overlooked. Unravelling how xylose affects regulation of metabolism, *i.e*. is xylose sensed as a fermentable carbon source and capable of catabolite repression like glucose, or is it rather sensed as a non-fermentable carbon source, is highly important in achieving understanding for engineering this yeast for efficient anaerobic fermentation of xylose. Thus, our aim was to determine how signalling and carbon catabolite repression differ in cells growing on either glucose or xylose. We carried out batch fermentations on these sugars under fully aerobic conditions to avoid the effect of oxygen limitation on the redox balance in cells grown on xylose. In previous transcriptome and proteome studies the metabolism on xylose was compared only with either glucose repressed [17] or derepressed cells [21], whereas comparison of xylose-grown cells with both glucose repressed and derepressed cells in the present study enabled responses solely due to the absence of glucose repression to be distinguished from those directly linked to the metabolism of xylose. The data reveal novel information on transcript and protein levels of xylose-metabolising cells indicating that they have both fermentative and respirative features. Importantly, although xylose was able to repress many of the glucose-repressible genes, repression was only partial compared with the glucose-grown and fully repressed cells. Additionally, signalling in the Snf1p/Mig1p glucose repression and Snf3p/Rgt2p-Rgt1p glucose induction pathways appeared different from that in the glucose-grown cells.

## Materials and methods

### Strains

Genetically modified *S. cerevisiae *strain VTT-C-99318 (CEN.PK2-1D *ura3*::*XYL1 XYL2 his3*::*XKS1 kanMX*) [22] was derived from CEN.PK2-1D (*MATα*, *leu2*-3/112, *ura3-52*, *trp1-289*, *his3Δ1*, *MAL2-8*^c^, *SUC*2) [23] and contains the genes *XYL1 *and *XYL2 *of *P. stipitis *encoding xylose reductase and xylitol dehydrogenase, respectively, integrated into the *URA3 *locus. *XYL1 *is expressed under a *PGK1 *promoter and *XYL2 *under a modified *ADH1 *promoter [24]. In addition, the strain contains an additional copy of the endogenous xylulokinase encoding gene of *S. cerevisiae *with the modified *ADH1 *promoter [24] and integrated into the *HIS3 *locus. The integration of *XYL1 *and *XYL2 *was described by Toivari *et al*., and that of *XKS1 *by Richard *et al*. [8,25].

### Aerobic batch fermentations

VTT-C-99318 was grown at 30°C in 1.8-litre bioreactors (Chemap CMF Bioreactor, Chemap AG, Volketswil, Switzerland) with a working volume of 1.5 litres. The pH was kept at 5.5 by automatic addition of 2 M KOH, and the stirrer speed at 500 rpm. Mass flow controllers (Bronkhorst High-Tech B.V, Ruurlo, Netherlands) regulated the total gas flow at 0.5 standard litres per minute (SLPM). The composition of the fermentor off-gas was analysed using a QMG 421C quadrupole mass spectrometer (Balzers Pfeiffer Scandinavia AB, Sweden) as described previously [14]. The medium was synthetic complete (SC) (modified from Sherman, 1983) [26]) supplemented with 0.5 ml silicone antifoam l^-1 ^(AnalaR BDH, UK) and 50 g l^-1 ^xylose or 50 g l^-1^glucose. Triplicate cultures (F0, F1, and F2) were carried out on both carbon sources. Cultures were started with an initial OD_600 _of 1 to 1.5 from pre-cultures grown for 20 h at 30°C on SC with 30 g/L glucose.

The 25 ml samples for transcriptome and proteome analyses were harvested by centrifugation (2 min, 5000 g) after 5 h and 24 h growth for glucose cultures and after 72 h growth for xylose cultures and frozen in liquid nitrogen. Cell growth was measured as OD_600 _and cell dry mass as described previously [14]. Extracellular concentrations of glucose, xylose, xylitol, ethanol, acetate and glycerol were analysed by high-performance liquid chromatography as described previously [14].

Samples from the F2 cultivations on each carbon source were hybridised three times on Affymetrix Yeast Genome S98 arrays (hybridisations denoted as H2.1, H2.2 and H2.3; see [Additional files 1 and 2]) and the samples from the cultivations F0 and F1 once (hybridisations denoted as H0 and H1, respectively; see [Additional files 1 and 2]). Proteome analyses were carried out with samples taken at the same time points as the samples for the transcriptome analyses.

### Affymetrix hybridisations, data acquisition and analysis

Affymetrix hybridisations and analyses were carried out according to the protocols provided by Affymetrix, Inc. Double-stranded cDNA was synthesised from 5 μg total RNA. An *in vitro *transcription (IVT) reaction was then carried out to produce biotin-labelled cRNA from the cDNA. The cRNA was fragmented and hybridised to a Test3 array for quality control and then to the Yeast Genome S98 array for 16 h at 45°C. The arrays were washed and stained with streptavidin-phycoerythrin in an Affymetrix Fluidics Station 400 and scanned (Agilent G2500A GeneArray Scanner). The data were extracted with Affymetrix MicroArray Suite 5.0 software.

Array data were pre-processed using the Robust Multichip Average (RMA) [27,28] implemented in the Bioconductor (version 1.5.0) [29] extension to the R software environment for statistical computing and graphics (version 2.0.1). The RMA-method computes log_2 _scale expression values from cell intensity files using the RMA model of probe-specific correction of perfect match (PM) probes and quantile normalisation [28]. A visual inspection of the scatter plots of the RMA pre-processed expression values, and the Pearson correlations between the replicate arrays did not indicate outlying arrays [Additional files 3, 4, 5].

After the pre-processing, TIGR MultiExperiment Viewer (version 3.1) was used to analyse the expression values [30]. The replicate values were averaged and genes which were differentially expressed between the glucose repressed and derepressed samples and the xylose samples were detected using one-way ANOVA at a *p*-value 0.01 based on 1000 permutation tests [31]. The expression values of significantly expressed genes (1439) were then mean centred, the biological replicate arrays were averaged and the values clustered using K-means with Euclidean distance [32]. Clustering was performed several times with varying parameters and the most optimal result was obtained with 13 clusters. Since the 13 clusters had 8 distinct expression profiles, and furthermore, as combining the clusters with similar expression profiles lowered the *p*-values of best gene ontology (GO) classes [33], the clusters were manually reorganised into 8 clusters.

### Two-dimensional gel electrophoresis of the proteome

5–10 mg dry mass of cells was suspended in 150 μl of 10% (v/v) trichloroacetic acid (TCA, Merck, USA) in a 1.5 ml microcentrifuge tube. 500 μl glass beads (0.5 mm diameter; Biospec Products, USA) were added and the tubes shaken in a MiniBeadbeater 8 (Biospec Products) at homogenisation speed three times for 30 seconds. The tubes were cooled on ice between each homogenisation step. The supernatants were collected and proteins were precipitated by adding 600 μl of -20°C acetone and incubating 30 min on ice. Precipitated proteins were collected by centrifugation for 30 min, 13 000 rpm, at 4°C, rinsed once with 600 μl of -20°C acetone, and re-suspended in 450 μl of 7 M urea (Promega, USA), 2 M thiourea (Fluka, USA), 4% (w/v) CHAPS (Fluka), 1% (w/v) Pharmalytes 3–10 (Pharmacia, Sweden) and 1% (w/v) DTT (Sigma) by gently shaking for 20 min at room temperature. Supernatants were collected by centrifugation for 5 min, 13 000 rpm. The protein concentration of the supernatants was determined by the Non-Interfering Protein Assay (Geno Technology, Inc., USA) and the samples were stored at -70°C prior to isoelectric focusing.

The isoelectric focusing and the second dimension on 11% (w/v) SDS-PAGE were carried out as described earlier [15]. After electrophoresis the gels were fixed for 1.5 h in 30% (v/v) ethanol and 0.5% (v/v) acetic acid, and stained either only with Sypro Ruby (Molecular Probes, USA) or with phosphoprotein-specific Pro-Q Diamond (Molecular Probes) followed by Sypro Ruby, according to instructions of the manufacturer. The stained gels were scanned with a resolution of 100 microns on a Typhoon instrument (GE Healthcare, USA). The gel images were analysed using the PDQuest software (Bio-Rad, USA). The gel patterns from different gels were automatically matched with some additional manual editing, and the quantities of matching spots in different gels were compared. For each condition, average spot quantities were calculated from two to four gels of each sample taken from each of the triplicate glucose and xylose cultivations. The quantification of the resolved proteins was normalised to the total optical density in each gel image. The data from 2-DE (two-dimensional electrophoresis) gels were analysed using the TIGR MultiExperiment Viewer (version 3.1) [30]. Proteins which had different abundance in glucose compared with xylose cultures were identified using the one-way ANOVA at a p-value of 0.01 [31]. The 220 protein-spot intensity values obtained were mean centred, the spots from replicate gels averaged, and the signal intensity values for identified protein spots (in total 70) were clustered using hierarchical clustering with Euclidean distance and average linkage [34].

The degree of phosphorylation was calculated by dividing the Pro-Q Diamond signal of a protein spot with the total protein amount of the spot determined by the Sypro Ruby signal. The averages and standard errors of the mean (SEM) were calculated from six replicate gels (2 replicate gels per sample from each of the three fermentations).

### Protein identification by mass spectrometry and proteome data analysis

Excised gel spots were destained in a freshly prepared solution of 30 mM potassium ferricyanide and 100 mM sodium thiosulfate (1:1) and then dehydrated in acetonitrile (ACN). For protein alkylation, the dried gel pieces were incubated in 5 mM tris (2-carboxyethyl) phosphine and 55 mM iodoacetamide (Sigma, USA) in dark at room temperature for 1 h, followed by washing with 100 mM ammonium hydrogen carbonate (NH_4_HCO_3_) and dehydration with ACN. The gel pieces were rehydrated in 10 to 15 μl 10 mM NH_4_HCO_3_, 10% (v/v) ACN containing 0.01 μg/μl sequencing grade trypsin (Promega, USA), and digested overnight at 37°C. To elute the tryptic peptides from the gel, the pieces were incubated twice in 150 μl 66% ACN (v/v) and 0.1% (v/v) trifluoroacetic acid (TFA; Fluka) for 30 min at 37°C. The peptide eluents were dried to a minimal amount of liquid in a SpeedVac, and suspended in 5 μl of 0.1% (v/v) TFA.

For co-crystallisation of peptides with the matrix, an aliquot of the peptide solution was mixed with a saturated matrix solution in a ratio of 1:10, and 1 to 2 μl was dispensed on a MALDI target and let to dry at room temperature. The matrix solution used was prepared by dissolving 17 mg α-cyano-4-hydroxycinnamic acid (Sigma) in 1 ml of 33% (v/v) ACN and 0.1% (v/v) TFA.

MALDI-TOF analyses were carried out with Autoflex (Bruker Daltonics, Bremen, Germany), equipped with a nitrogen pulsed laser (337 nm), operated in positive mode. Typically, mass spectra were acquired by accumulating spectra of 400 laser shots. External calibration was performed for molecular assignments using a peptide calibration standard (Bruker Daltonik GmbH, Leipzig, Germany).

Protein identifications were performed by searching the peptide masses against the National Center for Biotechnology Information (NCBI) non-redundant database using Prowl's ProFound – Peptide Mapping (Rockefeller University) [35], Protein Prospector ms-fit (University of California, San Francisco) [36] and Matrix Science's Mascot – Peptide Mass Fingerprint (Matrix Science Ltd, UK) [37]. Protein identifications by peptide mass fingerprinting were further evaluated by comparing the calculated and observed molecular masses and pI-values, as well as the number of peptides matched and the percentage of sequence coverage.

## Results

### Experimental design and data analysis of the transcriptome and proteome

Three aerobic batch fermentations were carried out both on 50 g l^-1 ^glucose and on 50 g l^-1 ^xylose to compare the yeast transcriptome and proteome of cells growing on xylose with that of glucose repressed and glucose derepressed cells. Samples of the xylose-grown cells were harvested at 72 h from the start of the xylose cultures with 32 ± 1 g l^-1 ^of residual xylose present. Samples of the glucose repressed cells were harvested at 5 h from the start of the glucose cultures with 37 ± 2 g l^-1 ^of residual glucose present. Samples of the glucose derepressed cells were harvested at 24 h from the start of the glucose cultures containing no glucose but 13 ± 1 g l^-1 ^of accumulated ethanol.

**Figure 1:**
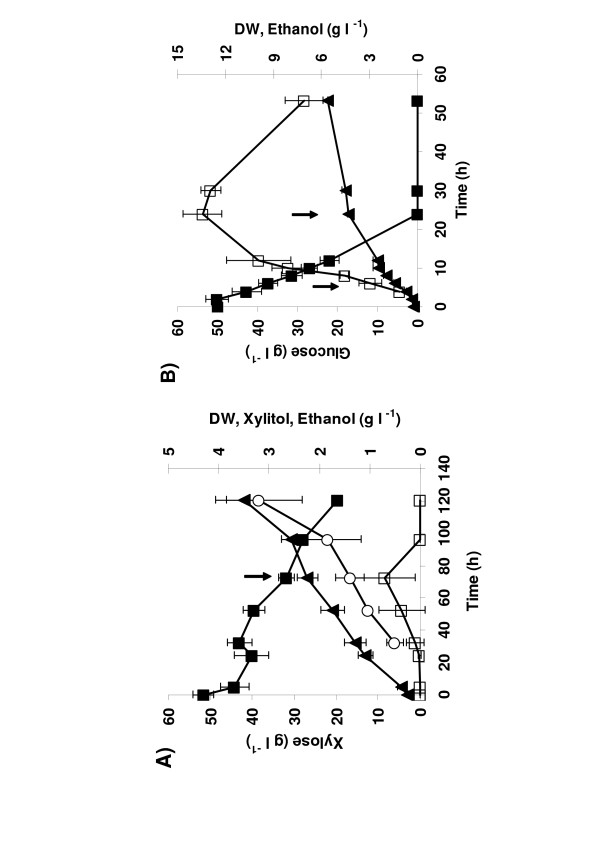
**Recombinant xylose-metabolising *S. cerevisiae *in aerobic batch cultures on xylose (A) and glucose (B) showing concentrations (g l^-1^) of A) xylose (■), cellular dry weight; DW (▲), ethanol (□) and xylitol (○) and B) glucose (■), cellular dry weight; DW (▲), and ethanol (□). **Cultures were maintained at pH 5.5, 30°C, 500 rpm, 0.33 volume air [volume culture]^-1 ^min^-1^. Data represent the average and standard deviation of three separate batch cultures on each carbon source. The arrows indicate times at which samples were taken for transcriptome and proteome analyses.

The volumetric consumption and production profiles of xylose, glucose, biomass, xylitol and ethanol (g l^-1^) of the cultures are shown in Figure 1. In addition, some acetate and glycerol (less than 2 g l^-1 ^each) were produced in the glucose cultures, and less than 4 g l^-1^acetate, 3 g l^-1 ^glycerol and 2 g l^-1 ^ethanol were measured in the xylose cultures. The doubling time of the cells in the xylose cultures was 28 ± 2 h (during 5 to 72 h), and in the glucose cultures 1.8 ± 0.1 h (during 2 to 5 h) and 73 ± 3 h (during 24 to 53 h).

**Figure 2:**
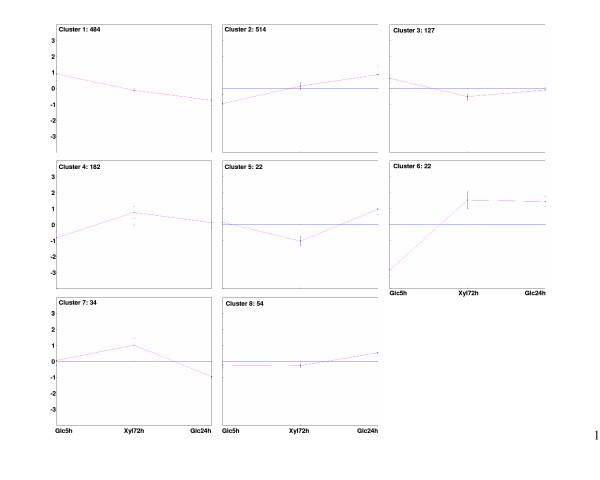
**The eight distinct clusters of the 1439 differentially expressed genes determined by K-means with Euclidean distance.** The y-axis corresponds to the difference of a gene relative to the mean expression of the gene in all samples on a log_2_-scale (values above zero-level represent up-regulation and below it down-regulation). The red lines represent the average expression pattern of each cluster. The x-axes are the 5 h and 24 h glucose and 72 h xylose samples (Glc5h, Glc24h and Xyl72h, respectively). The total number of genes in each cluster was: 484, 514, 127, 182, 22, 22, 34 and 54 for clusters 1 to 8, respectively.

The 1439 genes, which were expressed differently (based on ANOVA at a p-value 0.01) between the glucose repressed (Glc5h), glucose derepressed (Glc24h) and xylose (Xyl72h) samples were organised into eight separate clusters with distinct expression profiles shown in Figure 2. The top three gene ontology classes of each cluster and the number of genes found in each class are listed in Table 1. All genes in each cluster of Figure 2 are shown in additional files [Additional files 6, 7, 8, 9, 10, 11, 12, 13].

**Table 1:** The top three gene ontology (GO) classes in each of the eight clusters in Figure 1. *n *is the number of the genes with the specified function, % is the percentage of the genes in the cluster with the specified function, and *p*-value is the binomial distribution probability [33]. (Total number of genes in clusters 1 to 8: 484, 514, 127, 182, 22, 22, 34 and 54, respectively)

Cluster	GO-class	Annotation	*n*	%	*p*-value
1	GO:0009058	Biosynthesis	159	32.9	1.96E-23
	GO:0044249	Cellular biosynthesis	148	30.6	1.95E-21
	GO:0044238	Primary metabolism	286	59.1	5.51E-19
2	GO:0006091	Generation of precursor metabolites and energy	67	13.0	5.89E-25
	GO:0015980	Energy derivation by oxidation of organic compounds	57	11.1	6.24E-21
	GO:0006119	Oxidative phosphorylation	20	3.9	5.25E-11
3	GO:0006896	Golgi to vacuole transport	5	3.9	1.39E-05
	GO:0006892	Post-Golgi transport	6	4.7	2.10E-04
	GO:0042147	Retrograde transport, endosome to Golgi	3	2.4	6.40E-04
4	GO:0044242	Cellular lipid catabolism	2	1.1	4.99E-03
	GO:0016042	Lipid catabolism	2	1.1	4.99E-03
	GO:0006000	Fructose metabolism	2	1.1	4.99E-03
5	GO:0006790	Sulphur metabolism	6	27.3	8.16E-09
	GO:0000096	Sulphur amino acid metabolism	4	18.2	1.71E-06
	GO:0015837	Amine transport	4	18.2	8.44E-06
6	GO:0000017	Alpha-glucoside transport	2	9.1	1.00E-04
	GO:0042946	Glucoside transport	2	9.1	1.00E-04
	GO:0008643	Carbohydrate transport	2	9.1	4.21E-03
7	GO:0009082	Branched chain family amino acid biosynthesis	5	14.7	2.50E-09
	GO:0009081	Branched chain family amino acid metabolism	5	14.7	7.00E-09
	GO:0009098	Leucine biosynthesis	3	8.8	1.30E-06
8	GO:0000002	Mitochondrial genome maintenance	4	7.4	3.24E-05
	GO:0051294	Establishment of spindle orientation	2	3.7	1.66E-03
	GO:0051293	Establishment of spindle localization	2	3.7	1.66E-03

70 out of 547 protein spots separated on the 2-DE gels had different abundances between the glucose repressed (Glc5h), glucose derepressed (Glc24h) and xylose (Xyl72h) samples and were identified by MALDI-TOF peptide mass mapping. They represented 55 different proteins, with 12 proteins present as multiple protein spots on the gels (Table 2) [Additional files 14 and 15]. 73% of the responses at the protein level correlated qualitatively with the microarray data (Table 2).

**Table 2:** Proteins which were differentially translated in the glucose repressed, glucose derepressed and xylose-grown cells, identified from the 2-DE gels [Additional files 14 and 15].

				log2 X72/G24		log2 X72/G5		log2 G24/G5	
**Swiss Prot Id**	**Protein^a^**	**ORF**	**Function**	2D	array	2D	array	2D	array

P32316	**Ach1p**	YBL015W	acetyl-CoA hydrolase	0.0	-1.0	3.4	2.7	3.4	3.7
P00330	Adh1p_a	YOL086C	alcohol dehydrogenase	1.4	0.9	0.2	0.3	-1.2	-0.6
P00330	Adh1p_b	YOL086C	alcohol dehydrogenase	2.1	0.9	0.5	0.3	-1.6	-0.6
P00331	**Adh2p**	YMR303C	alcohol dehydrogenase	4.8	-2.6	5.7	1.4	0.9	4.0
P47143	**Ado1p_a**	YJR105W	adenosine kinase	-1.1	0.4	-1.4	-1.3	-0.2	-1.7
P47143	**Ado1p_b**	YJR105W	adenosine kinase	-1.4	0.4	-1.9	-1.3	-0.5	-1.7
P38013	Ahp1p	YLR109W	thioredoxin peroxidase	-0.4	0.0	0.5	-0.2	1.0	-0.2
P54114	**Ald3p**	YMR169C	aldehyde dehydrogenase	0.4	0.8	1.8	1.8	1.4	1.0
P46367	Ald4p	YOR374W	aldehyde dehydrogenase (NAD^+^)	1.7	-0.2	3.6	1.0	1.9	1.1
P54115	**Ald6p_a**	YPL061W	aldehyde dehydrogenase (NADP^+^)	-0.9	-2.6	0.2	-1.9	1.0	0.7
P54115	**Ald6p_b**	YPL061W	aldehyde dehydrogenase (NADP^+^)	-2.0	-2.6	-2.8	-1.9	-0.8	0.7
P28777	**Aro2p**	YGL148W	chorismate synthase	-0.7	0.4	-1.4	-1.4	-0.7	-1.8
P53090	**Aro8p**	YGL202W	aromatic-amino-acid transaminase	-1.7	1.5	-0.6	-1.0	1.1	-2.5
P38011	**Asc1p**	YMR116C	molecular function unknown	-0.6	1.8	-2.4	-1.1	-1.8	-3.0
P07251	Atp1p	YBL099W	hydrogen-transporting ATP synthase	0.1	-0.2	1.8	0.7	1.7	0.9
P00830	Atp2p	YJR121W	hydrogen-transporting ATP synthase	0.2	-0.4	1.6	0.9	1.4	1.2
P38891	**Bat1p**	YHR208W	branched-chain-amino-acid transaminase	1.9	3.9	0.4	0.7	-1.5	-3.2
P07256	**Cor1p_a**	YBL045C	ubiquinol-cytochrome-c reductase	0.6	-0.2	1.3	1.0	0.8	1.2
P07256	**Cor1p_b**	YBL045C	ubiquinol-cytochrome-c reductase	0.3	-0.2	1.3	1.0	1.0	1.2
P07256	**Cor1p_c**	YBL045C	ubiquinol-cytochrome-c reductase	-0.1	-0.2	1.3	1.0	1.5	1.2
P06115	**Ctt1p**	YGR088W	catalase	1.4	1.8	3.4	2.4	2.0	0.6
P31373	Cys3p	YAL012W	cystathionine gamma-lyase	-2.3	-0.6	-2.9	-3.1	-0.6	-2.4
P32582	Cys4p_a	YGR155W	cystathionine beta-synthase	-2.5	-0.8	-0.2	-1.1	2.3	-0.3
P32582	Cys4p_b	YGR155W	cystathionine beta-synthase	-0.9	-0.8	-1.7	-1.1	-0.9	-0.3
P06634	Ded1p	YOR204W	RNA helicase	0.5	0.3	-2.2	0.5	-2.7	0.2
P14306	**Dka1p**	YLR178C	carboxypeptidase Y inhibitor	-1.2	-0.6	1.7	0.8	2.9	1.4
P39976	Dld3p	YEL071W	D-lactate dehydrogenase (cytochrome)	-2.0	-0.2	-2.4	-0.6	-0.3	-0.4
P00924	Eno1p_a	YGR254W	phosphopyruvate hydratase	1.5	0.6	5.2	0.5	3.7	-0.1
P00924	Eno1p_b	YGR254W	phosphopyruvate hydratase	3.5	0.6	5.7	0.5	2.2	-0.1
P00924	Eno1p_c	YGR254W	phosphopyruvate hydratase	1.2	0.6	2.6	0.5	1.3	-0.1
P14065	**Gcy1p**	YOR120W	aldo-keto reductase	2.0	0.3	3.7	3.1	1.7	2.8
Q00055	Gpd1p	YDL022W	glycerol-3-phosphate dehydrogenase	0.7	0.0	1.9	0.2	1.2	0.2
P00950	Gpm1p	YKL152C	phosphoglycerate mutase	1.7	0.5	1.6	-0.1	0.0	-0.7
P38625	**Gua1p**	YMR217W	GMP synthase	-0.8	1.3	-1.8	-1.0	-1.0	-2.3
P15454	**Guk1p**	YDR454C	guanylate kinase	-1.7	0.3	-1.4	-1.3	0.3	-1.6
P15992	Hsp26p_a	YBR072W	heat shock protein	0.1	-0.2	3.0	2.0	2.9	2.2
P15992	Hsp26p_b	YBR072W	heat shock protein	-0.6	-0.2	2.2	2.0	2.8	2.2
P04807	**Hxk2p_a**	YGL253W	hexokinase	2.6	2.9	10.7	-0.5	8.1	-3.4
P04807	**Hxk2p_b**	YGL253W	hexokinase	0.7	2.9	-1.1	-0.5	-1.8	-3.4
P28241	**Idh2p**	YOR136W	isocitrate dehydrogenase (NAD+)	-0.9	-0.5	0.7	0.2	1.7	0.7
P00817	Ipp1p	YBR011C	inorganic diphosphatase	-1.7	-0.5	-1.5	-0.9	0.3	-0.5
P53598	Lsc1p	YOR142W	succinate-CoA ligase (ADP-forming)	-0.5	-0.4	0.6	0.1	1.1	0.4
P36060	Mcr1p	YKL150W	cytochrome-b5 reductase	1.8	0.1	3.3	1.5	1.4	1.4
P05694	Met6p	YER091C	methionine synthase	-2.5	-1.3	-2.9	-1.0	-0.4	0.3
P04147	Pab1p	YER165W	poly(A) binding protein	0.4	0.2	-1.1	-0.3	-1.6	-0.5
P06169	**Pdc1p_a**^b^	YLR044C	pyruvate decarboxylase	-1.7	2.0	-1.8	-0.2	0.0	-2.2
P06169	**Pdc1p_b**^b^	YLR044C	pyruvate decarboxylase	-2.1	2.0	-1.2	-0.2	0.9	-2.2
P17967	**Pdi1p**	YCL043C	protein disulfide isomerase	-0.7	-0.1	-2.6	-0.5	-1.9	-0.4
P00560	Pgk1p_a	YCR012W	phosphoglycerate kinase	1.1	0.6	1.8	0.1	0.7	-0.5
P00560	Pgk1p_b	YCR012W	phosphoglycerate kinase	1.9	0.6	2.6	0.1	0.7	-0.5
P34227	Prx1p	YBL064C	thioredoxin peroxidase	-0.2	-0.9	1.7	0.7	1.9	1.6
Q12335	Pst2p	YDR032C	molecular function unknown	-0.6	-0.1	0.8	-0.4	1.5	-0.3
P07703	**Rpc5p**	YPR110C	DNA-directed RNA polymerase	-0.3	0.5	-1.5	-1.3	-1.2	-1.8
P26783	Rps5p	YJR123W	structural constituent of ribosome	-0.6	0.7	-3.0	-0.6	-2.4	-1.2
P19358	**Sam2p**	YDR502C	methionine adenosyltransferase	-2.2	-2.0	-2.5	-2.6	-0.3	-0.6
P07283	**Sec53p_a**	YFL045C	phosphomannomutase	-0.1	0.2	-2.5	-1.6	-2.4	-1.8
P07283	**Sec53p_b**	YFL045C	phosphomannomutase	-1.1	0.2	-2.0	-1.6	-0.8	-1.8
P33330	**Ser1p**	YOR184W	phosphoserine transaminase	-0.8	-0.3	-0.9	-1.0	-0.1	-0.8
P37291	Shm2p	YLR058C	glycine hydroxymethyltransferase	0.1	-0.1	-1.5	0.6	-1.6	0.7
Q03144	Sno1p	YMR095C	molecular function unknown	0.3	0.4	-0.8	2.0	-1.1	1.6
P00447	Sod2p	YHR008C	manganese superoxide dismutase	-0.5	-0.2	1.9	1.1	2.4	1.2
P15705	Sti1p	YOR027W	Hsp90 cochaperone	0.6	0.2	1.7	0.3	1.1	0.1
P23254	**Tkl1p**	YPR074C	transketolase	-0.6	1.1	-1.0	-0.6	-0.5	-1.7
P00942	Tpi1p	YDR050C	triose-phosphate isomerase	-1.1	0.2	0.5	-0.1	1.5	-0.3
P17649	Uga1p	YGR019W	4-aminobutyrate transaminase	-0.8	-0.1	1.4	0.2	2.2	0.3
Q12363	Wtm1p_a	YOR230W	transcriptional modulator	0.1	-0.1	1.7	0.2	1.6	0.3
Q12363	Wtm1p_b	YOR230W	transcriptional modulator	-0.1	-0.1	1.3	0.2	1.4	0.3
P23180	Yhl021p	YHL021C	molecular function unknown	-0.5	-0.8	1.6	0.6	2.1	1.3
P35691	**Ykl056cp**	YKL056C	molecular function unknown	-1.0	0.5	-1.8	-1.4	-0.9	-1.9
Q04869	**Ymr315wp**	YMR315W	molecular function unknown	2.5	2.3	3.1	1.8	0.6	-0.4

Log2 values of the protein abundance ratios, and the expression ratios for the corresponding gene of cells grown on xylose for 72 h (X72) to cells grown on glucose for 24 h (G24), or 5 h (G5), and of cells grown on glucose for 24 h to cells grown on glucose for 5 h are given. Proteins whose encoding genes showed a statistically significant expression difference in the samples studied are marked with bold text.

^a^Different pI forms of a protein indicated by a, b and c

^b^These spots of Pdc1p had substantially lower molecular weight compared with the major Pdc1p protein spot identified earlier [15] and are likely either degraded or post-translationally processed forms of the enzyme.

### Carbon source sensing and signalling in cells metabolising xylose

One of the first responses of *S. cerevisiae *to glucose is the induction of genes encoding hexose transporters (Hxt) that are also used for xylose uptake [38,39]. The signal for the presence of glucose is mediated via the glucose sensor proteins Snf3p and Rgt2p, responding to low and high glucose concentrations, respectively [40,41]. The expression of *SNF3 *is repressed at high concentrations of glucose via Mig1p ([42] and references therein). In the xylose-grown cells, the transcript levels of *SNF3 *were higher than in the glucose repressed cells, but lower than in the glucose derepressed cells (Fig. 2, cluster 2), while *RGT2 *had its highest expression in the xylose-grown cells (Fig. 2, cluster 4). The expression of *RGT2 *is reported to be independent of glucose concentration [43], but in the present study derepression was observed (Fig. 2, cluster 4).

*RGT1*, encoding a transcription factor of *HXT *genes had, like *SNF3*, higher transcript levels in the xylose-grown cells than in the glucose repressed cells, but lower than in the glucose derepressed cells (Fig. 2, cluster 2). The expression of *RGT1 *is reported not to be regulated in response to glucose ([42] and references therein), but the expression profile in cluster 2 suggests that its expression was derepressed in the absence of glucose, and to a lesser extent also on xylose (Fig. 2). Consistently, *HXT2 *and *HXT3*, which are repressed by *RGT1*, had highest expression in the presence of glucose and lowest in the glucose derepressed cells (Fig. 2, cluster 1). In addition to its function in repressing *HXT *genes in the absence of glucose [44], Rgt1p is required for repression of *HXK2 *at low levels of glucose together with the transcription cofactor Med8p [45]. The expression of *RGT1 *and *HXK2 *was as expected: while *RGT1 *was expressed at its highest level in the glucose derepressed cells, *HXK2 *had its lowest expression in these cells (Fig. 2, cluster 1). *MTH1 *encoding a corepressor of *RGT1 *had its highest expression in the xylose-grown cells (Fig. 2, cluster 4). Mth1p is involved in maintaining the repression of *HXT *genes when glucose is not present [46]. Nevertheless, in the cells grown on xylose, *HXT2 *and *HXT3 *had higher transcript levels compared with the glucose derepressed cells (Fig. 2, cluster 1), *HXT6 *compared with the glucose repressed cells (Fig. 2, cluster 2), and *HXT4 *and *HXT16 *compared with both the glucose repressed and derepressed cells (Fig. 2, clusters 7 and 4, respectively).

### The metabolism of the recombinant *S. cerevisiae *on xylose appears neither as fully glucose repressed nor as fully derepressed

In xylose-grown cells, the genes encoding activities of the TCA and glyoxylate cycles, respiration and gluconeogenesis were expressed more strongly than in glucose repressed cells but less strongly than in derepressed cells (Fig. 2, cluster 2). On glucose these genes are repressed via the pathway involving Snf1p, Mig1p and Hxk2p [47]. In more detail, cluster 2 contained genes encoding enzymes of respiration (*e.g. COX4*, *COX5a*, *COX7*, *COX14-15*, *COR1*, *QCR2*, *QCR7*, *QCR9*, *ATP3-5*, *ATP16*), gluconeogenesis (*e.g. FBP1*, *PCK1*), the TCA and glyoxylate cycles (*e.g. KGD1-2*, *SDH1-4*, *FUM1*, *MDH1*, *CIT1-3*, *MLS1*, *ICL1*), alcohol catabolism (*e.g. ADH2*), and trehalose and glycogen synthesis (*e.g. TSL1*, *GLC1-3*, *GSY2*) (Table 1). In addition, genes encoding transcriptional regulators of the aforementioned genes, such as *ADR1*, *CAT8*, *HAP4*, *SIP1-2 *and *4*, and *REG2 *[48] were present in cluster 2 (Fig. 2). In the proteome analysis of the corresponding samples, a similar trend was seen in the abundance of proteins with respiratory functions, such as ATP synthases Atp1p and Atp2p, ubiquinol-cytochrome-c reductase (Cor1p; two isoforms detected), isocitrate dehydrogenase Idh2p (expression of *IDH2 *was comparable in the glucose repressed and xylose-grown cells; Fig. 2, cluster 8), and the α-subunit of succinyl-CoA ligase Lsc1p (Table 2) [Additional file 15].

Distinct profiles in the abundance of transcripts and enzyme levels of the glycolytic and ethanol pathways were observed. *HXK2 *and *PGI1 *had lower expression in the xylose-grown cells compared with the glucose repressed cells (Fig. 2, cluster 1). On the other hand, the xylose-grown cells had higher amounts of Pgk1p, Gpm1p and Adh1p, compared with the glucose repressed cells (Table 2) [Additional file 15]. In addition, Eno1p and Adh2p (whose encoding gene had higher transcript abundance on xylose compared with the glucose repressed cells; Fig. 2, cluster 2) were most abundant in the xylose-grown cells (Table 2) [Additional file 15]. The latter two proteins are typically synthesised on non-fermentable carbon sources [49,50].

**Figure 3:**
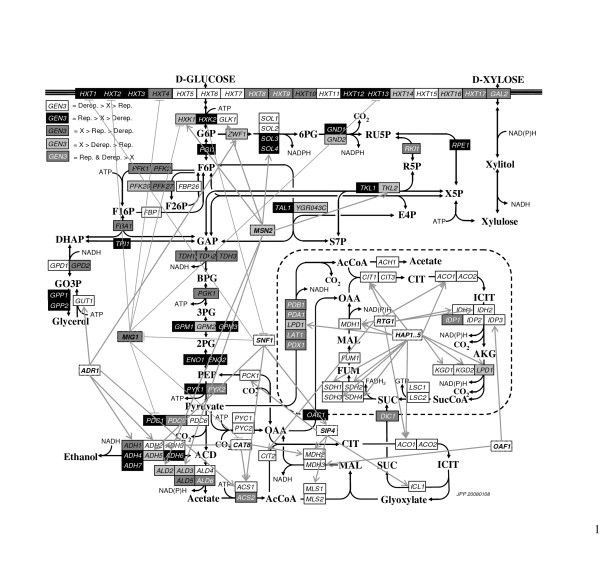
**Expression profiles of genes involved in the main metabolic networks of *S. cerevisiae*. **Transcription factors are presented with boldfacing and connected to the respective target genes with gray lines. The connections shown between transcription factors and their target genes are based on compilations in Yeast Proteome Database [70][80] and on the transcription factor binding network by Young and co-workers [82]. Expression of the genes presented in white boxes with black text was highest in the glucose derepressed cells (Derep.) and lowest in the glucose repressed cells (Rep.). Expression of the genes presented in black boxes with white text was highest in the glucose repressed cells and lowest in the glucose derepressed cells. Expression of the genes presented in dark gray boxes with black text was highest in the xylose-grown cells and lowest in the glucose derepressed cells. Expression of the genes presented in light gray boxes with black text was highest in the xylose-grown cells and lowest in the glucose repressed cells. Expression of the genes presented in gray boxes with white text was lowest in the xylose-grown cells. In addition to the genes shown in the figure, 89% of the genes (31 out of 35) annotated to GO category "Oxidative phosphorylation" and its daughter categories [33] had highest expression in the glucose derepressed cells, lowest expression in the glucose repressed cells and intermediate expression in the cells grown on xylose (data not shown).

Overlaying the comparison of gene expression in the glucose repressed, derepressed and xylose-grown cells on the main metabolic network (Fig. 3) further demonstrates how the genes encoding activities of the TCA and glyoxylate cycles had higher expression profiles on xylose compared to the glucose repressed cells, whereas the opposite was observed with many genes of glycolysis and the PPP. The differences indicated in the expression of all individual genes in Figure 3 were not statistically significant based on ANOVA, however, the analysis clearly illustrates the distinct trends in the expression of genes of individual pathways in the central carbon metabolism on xylose compared to the glucose repressed and derepressed cells.

In addition to Snf3p and Rgt2p, extracellular glucose is sensed via Gpr1p, which signals the presence of glucose via Gpa2p to the cyclic AMP-protein kinase A (cAMP-PKA) signalling pathway [51]. The glucose-induced activation of cAMP synthesis is subsequently repressed by glucose and therefore not considered to be operative during growth on glucose [52]. Most genes encoding components of the cAMP-PKA pathway had intermediate expression in the xylose-grown cells with highest expression in the glucose derepressed cells and lowest in the glucose repressed cells [Additional file 16]. Consistently, 52% of the genes (28/54 genes) repressed via this pathway in the presence of glucose had the same intermediate expression profile in the xylose-grown cells (data not shown).

### Some glucose repressible genes had their highest expression on xylose

*HXK1*, *HXT16 *and *SUC2*, which are all repressed by glucose via the Snf1p/Mig1p-pathway [42,53-55], had their highest expression levels in the xylose-grown cells (Fig. 2, cluster 4). *MAL11*, *MAL31*, and *MAL32*, encoding activities for the utilisation of maltose and representing classical prototype genes of glucose repression by Mig1p, were also relieved from glucose repression (Fig. 2, cluster 6). This was observed despite the fact that *GAL83 *encoding one of the β-subunits of the Snf1 kinase complex and enabling nuclear localisation of the kinase in the presence of non-fermentable carbon sources [56] had its lowest expression in the cells grown on xylose (Fig. 2, cluster 3), as if xylose was perceived as a fermentable, repressive carbon source.

### Growth on xylose affected expression of genes in the pathways of acetyl-CoA synthesis and consumption

The metabolic pathway responsible for the cytoplasmic synthesis of acetyl-CoA from ethanol involves alcohol dehydrogenase 2, cytoplasmic aldehyde dehydrogenase 6, and acetyl-CoA synthase encoded by *ADH2*, *ALD6 *and *ACS1*, respectively. *ADH2 *and *ACS1*, and the genes encoding the two transcription factors, Adr1p and Cat8p, which regulate their expression [57] had higher transcript levels on xylose compared to the glucose repressed cells (Fig. 2, cluster 2). However, expression of *ALD6 *was lower on xylose compared with the glucose repressed and derepressed cells (Fig. 2, cluster 5), as was the abundance of two isoforms of Ald6p in the proteome analysis (Table 2) [Additional file 15]. *ALD5 *encoding a mitochondrial, NADPH-dependent isoform of acetaldehyde dehydrogenase [58], was up-regulated on xylose (Fig. 2, cluster 7). In the proteome analysis, Ald4p, another mitochondrial, NADPH-dependent isoform, had its highest abundance on xylose (Table 2) [Additional file 15]. The expression of glucose repressed *ACH1*, encoding a mitochondrial acetyl-CoA hydrolase [59], had higher transcription on xylose compared to the glucose repressed cells (Fig. 2, cluster 2), which was also reflected in the abundance of Ach1p (Table 2) [Additional file 15].

### Distinct phosphorylation patterns of some major glycolytic enzymes on xylose and glucose

**Figure 4:**
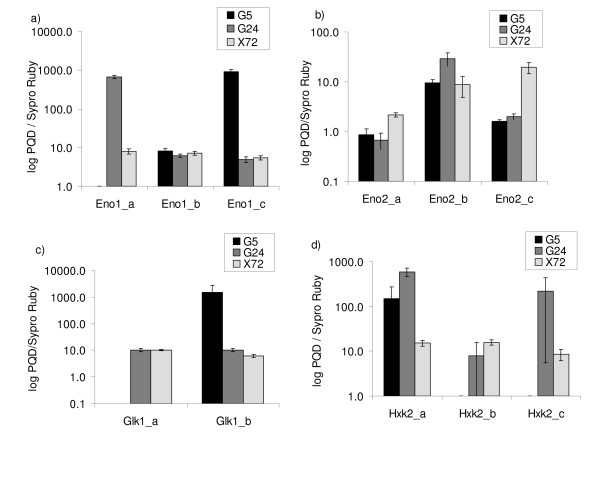
**Log-scale ratio of intensity of Pro-Q Diamond phosphoprotein stain to Sypro Ruby signal (total protein amount) of phosphorylated a) enolase 1, b) enolase 2, c) glucokinase, and d) hexokinase 2 protein isoforms identified from 2-DE gel analyses (a to c refer to different isoforms on gels). **G5 = cells after 5 h on glucose; G24 = cells after 24 h on glucose; X72 = cells after 72 h on xylose.

Interestingly, several glycolytic enzymes (*i.e*. Hxk2p, Pgk1p, Eno1p, Eno2p) had post-translationally modified forms with different abundances in the xylose-grown and glucose repressed and derepressed cells (Table 2) [Additional files 14, 15 and 17]. The fluorescent phosphoprotein-specific Pro-Q Diamond stain revealed reproducible, distinct patterns of phosphorylated forms of Hxk2p, Glk1p, Eno1p and Eno2p that were present in different relative quantities in the three different conditions studied (normalised to the total amount of each protein; Fig. 4) [Additional file 17]. Both Eno1p and Eno2p had three phosphorylated pI forms with different abundances in the xylose- and glucose grown cells (Fig. 4). The expression of *ENO1 *was expected to be repressed by glucose, but both *ENO1 *and *ENO2 *should be expressed during growth on non-fermentable carbon sources [49]. Two phosphorylated isoforms were identified for Glk1p and three for Hxk2p (Fig. 4). *GLK1 *should be expressed during growth on non-fermentable carbon sources and Hxk2p should be dominant during growth on glucose [55]. The expression of *ENO1 *and *GLK1 *did not significantly change between the three samples studied.

## Discussion

Transcription of a number of genes involved in the utilisation of alternative or non-fermentative carbon sources are repressed in the presence of glucose and derepressed in its absence [60]. Glucose sensing, signalling, and repression are thus closely linked to respirative and fermentative modes of metabolism. Accordingly, the way the cell recognises, senses, and signals the presence of xylose must affect the efficiency of its utilisation and fermentation. Previous studies of xylose-metabolising *S. cerevisiae *have suggested that xylose has a repressing effect on gene expression [21,61,62]. The results of the present study show that the expression of several genes repressed via the Snf1p/Mig1p glucose repression pathway *i.e*. genes encoding respiratory, TCA, glyoxylate cycle and gluconeogenic enzymes was lower during growth on xylose than in derepressed glucose-grown cells (24 h in Fig. 1). However, expression of these genes was higher than observed in glucose repressed cells (5 h in Fig. 1). On the other hand, some genes of the PPP and glycolysis and those typically expressed on non-fermentable carbon sources (*e.g. HXK1*, *SUC2*, *PFK26 *and some *MAL*-genes) showed increased expression in the cells growing on xylose. Thus, during xylose metabolism a mixed profile of gene expression was observed, suggesting that xylose does not activate all the glucose repression pathways in the same way as glucose.

The mechanisms of signal transduction by xylose are still unknown. It has been suggested that some hexose or triose phosphates derived from glycolysis [63,64] are involved in the regulation of gene expression, at least in lower glycolysis. The cellular concentration of hexose phosphates depends on the rate of glycolysis [65], but a correlation between the repression of genes and the concentration of hexose or triose phosphates on different carbon sources has not been shown [62]. When xylose is the main carbon source glycolytic flux to pyruvate is lower than for cells growing on glucose [14,66]. This may contribute to the observed higher expression of genes repressed via Snf1p/Mig1p in the xylose-grown cells, as Mig1p is expected to be totally dephosphorylated (and thus to repress its target genes) only at high glycolytic rates [67].

In recent studies, growth rate has been shown to affect the levels of transcripts, proteins and metabolites [68,69]. It could thus be argued that the intermediate expression level of many glucose repressed genes in the xylose-grown cells, compared with the glucose repressed and derepressed cells, is due to the different growth rate on xylose. Comparison of the present data with the data of growth rate regulated genes and proteins described in studies of Regenberg *et al*., 2006 and Castrillo *et al*., 2007, showed, however, that a majority (roughly 90%) of these genes and proteins (in clusters 1 and 2, Fig. 2) were with unknown function or associated with processes such as translation, RNA metabolism, ribosome biogenesis, amino acid metabolism, and stress, and not with the central pathways of carbon metabolism or glucose sensing and repression [68,69]. Additionally, in our previous chemostat studies, TCA cycle genes had a lower expression level on a mixture of xylose and glucose (27 and 3 g l^-1^, respectively) than under glucose limitation (10 g l^-1^) at the same specific growth rate suggesting that xylose had a repressive effect on the expression of these genes [21]. This supports the assumption that the lower growth rate is not the sole explanation for the only partial repression of the glucose repressed genes on xylose. The intermediate expression on xylose of respiratory, TCA and glyoxylate cycle genes was probably neither due to the lower concentration of ethanol on xylose compared with the glucose derepressed cells since Belinchón and Gancedo, (2003) showed that in a recombinant, xylose-metabolising *S. cerevisiae *strain, even in the presence of ethanol, xylose decreased the expression of *ICL1 *and *FBP1 *compared with derepressed, ethanol-grown cells [62]. Contradictorily, Jin *et al*., (2004), comparing the transcriptome of xylose-grown cells to that of glucose repressed cells, stated that xylose is not a repressive carbon source [17]. Their study, however, lacked the comparison of xylose-grown cells to fully derepressed cells, whereas comparison of the xylose-grown cells to the derepressed cells in the present study showed that xylose decreased the expression of several genes repressed by glucose via the Snf1p/Mig1p-pathway.

Yet another possibility for the intermediate expression in xylose-grown cells may be the way cAMP-PKA mediated pathways function on xylose. In the present study, over 50% of the genes repressible via this pathway [70] were expressed in xylose-grown cells at levels lower than those in glucose derepressed cells but above those in glucose repressed cells [Additional file 16]. Glucose is sensed by the receptor Gpr1p that initiates a signalling cascade leading to stimulation of fermentation. In the study of Rolland *et. al*., (2000) xylose (100 mM) was not sensed by Gpr1p [71]. In the present work, xylose was 210 mM at the time of sampling, and possibly even a higher concentration is needed for Gpr1p to sense this sugar. Furthermore, full activation of cAMP synthesis by glucose requires phosphorylation of glucose by Hxk1p, Hxk2p or Glk1p [71], which does not occur on xylose.

Glucose is also sensed at high and low concentrations, respectively by Rgt2p and Snf3p sensors. In glucose-grown cells the signal leads to induction of the *HXT *genes encoding glucose transporters by relieve of repression by Rgt1p through degradation of its co-repressors Mth1p and Std1p [43,72]. The Snf1p/Mig1p repression pathway contributes to glucose induction of *HXT *genes by repressing *SNF3 *and *MTH1 *[46]. The induction of *MTH1 *and only partial repression of *SNF3 *in the xylose-grown cells shows again that the glucose repression pathway via Snf1p/Mig1p does not operate to the same extent on xylose as it does on glucose. However, the expression profile of *SNF3 *and the highest expression of *RGT2 *in xylose-grown cells suggest that these sensors detect extracellular xylose.

Changes were observed in the expression of genes of acetaldehyde and acetyl-CoA metabolism. The increased expression of *ACS1 *and *ALD5*, and increased abundance of Ald4p suggest that acetaldehyde was utilised for growth instead of ethanol production during metabolism of xylose. The observed higher expression of *ACH1*, encoding the glucose repressed mitochondrial acetyl-CoA hydrolase, suggests that mitochondrial acetate concentration was perhaps elevated in xylose-grown cells compared with the glucose repressed cells. The changes may also reflect regulation of intracellular redox balance between the cytosol and mitochondria, since oxidation of acetaldehyde to acetate by Ald6p or Ald4/5p provides cytosolic and mitochondrial NADPH, respectively [73].

Jin *et al*., (2004) observed no change in the expression levels of genes encoding glycolytic enzymes in a xylose-utilising *S. cerevisiae *strain when xylose was provided as a carbon source instead of glucose in shake flask cultures [17]. In the present study, some of these genes had increased expression, and the proteome showed higher abundance of several glycolytic enzymes on xylose compared with either the glucose repressed or derepressed cells. Several recent studies suggest that regulation of glycolysis in *S. cerevisiae *occurs mainly at post-translational levels at least when aerobic and anaerobic conditions are compared, or ethanol and acetate are supplied instead of glucose [74-76]. Supporting these findings, the proteome analysis of the present study revealed that the relative abundance of different isoforms of some glycolytic enzymes correlated with the carbon source. Distinct patterns of phosphorylation of hexokinase 2, glucokinase and enolase in cells grown on xylose or glucose suggested that these enzymes were regulated differently by phosphorylation in the glucose repressed and derepressed cells, and in the cells grown on xylose. Apart from Hxk2p, pyruvate kinase (Pyk1/2p), and the regulatory Pfk26/27p [77,78] there is little information on the phosphorylation of glycolytic enzymes. It has been shown that Hxk2p is dephosphorylated on fermentable carbon sources, whereas both phosphorylated and dephosphorylated forms exist on poorly fermentable carbon sources [79]. The present data showed that only one of the three phosphorylated forms of Hxk2p was present in glucose repressed cells whereas the other two were present only in glucose derepressed cells and cells grown on xylose. This suggests that xylose was sensed rather as a non-fermentable carbon source. It is tempting to speculate that phosphorylation of also other glycolytic enzymes than the three kinases would be a yet poorly understood means to modulate the activity of glycolysis. However, phosphorylation may also relate to the role of these proteins in other cellular processes [80].

The present study shows that several genes that are subject to glucose repression were expressed during batch growth on xylose at levels intermediate between those in glucose repressed and derepressed cells. This suggests that cells growing on xylose do not have the appropriate expression levels of certain genes that may be one reason why they do not ferment xylose as fast as glucose. The results are consistent with the hypothesis that xylose does not activate all the glucose repression pathways in the same way as glucose, clearly indicating that this is an interesting area for further research towards improving xylose fermentation.

## Competing interests

The authors declare that they have no competing interests.

## Authors' contributions

LS participated in the design of the study, performed the batch fermentations, RNA isolations and 2-D gel based proteome analyses, analysed the data and wrote the paper together with LR. MK carried out the statistical analysis of transcription and proteome data. RS carried out the mass spectrometric identification of proteins. JPP carried out the analysis presented in Figure 3 and commented the paper. MP participated in the design of the study. LR participated in the design of the study and wrote the paper together with LS. All authors have read and approved the final manuscript.

## Supplementary Material

Additional file 1 Pearson correlation coefficient values between the biological and technical replicate arrays from samples of glucose fermentations. The data provided shows the Pearson correlation coefficient values between the biological and technical replicate arrays of from samples of glucose fermentations (Glc5h and Glc24h). Click here for file

Additional file 2 Pearson correlation coefficient values between the biological and technical replicate arrays of samples from xylose fermentations. The data provided shows the Pearson correlation coefficient values between the biological and technical replicate arrays of xylose samples from fermentations (Xyl72h). Click here for file

Additional file 3 Scatterplots of RMA pre-processed arrays from cells grown on glucose for 5 h. The figure provided represents the scatterplots of the expression values of the replicate microarrays hybridised with the samples derived from cells grown on glucose for 5 h. Click here for file

Additional file 4 Scatterplots of RMA pre-processed arrays from cells grown on glucose for 24 h. The figure provided represents the scatterplots of the expression values of the replicate microarrays hybridised with the samples derived from cells grown on glucose for 24 h. Click here for file

Additional file 5 Scatterplots of RMA pre-processed arrays from cells grown on xylose for 72 h. The figure provided represents the scatterplots of the expression values of the replicate microarrays hybridised with the samples derived from cells grown on xylose for 72 h. Click here for file

Additional file 6 Cluster 1. List of open reading frames in cluster 1 shown in Fig. 2 of the paper. Click here for file

Additional file 7 Cluster 2. List of open reading frames in cluster 2 shown in Fig. 2 of the paper. Click here for file

Additional file 8 Cluster 3. List of open reading frames in cluster 3 shown in Fig. 2 of the paper. Click here for file

Additional file 9 Cluster 4. List of open reading frames of in cluster 4 shown in Fig. 2 of the paper. Click here for file

Additional file 10 Cluster 5. List of open reading frames in cluster 5 shown in Fig. 2 of the paper. Click here for file

Additional file 11 Cluster 6. List of open reading frames of in cluster 6 shown in Fig. 2 of the paper. Click here for file

Additional file 12 Cluster 7. List of open reading frames in cluster 7 shown in Fig. 2 of the paper. Click here for file

Additional file 13 Cluster 8. List of open reading frames in cluster 8 shown in Fig. 2 of the paper. Click here for file

Additional file 14 Image of the 11% SDS-PAGE 2-DE-gel. The image of the 2-DE-gel showing the locations of the seventy protein spots, which had different abundance in cells growing on glucose or xylose. Click here for file

Additional file 15 Clustering of the proteome data. The figure shows seventy proteins, which were differentially translated in the glucose repressed, glucose derepressed and xylose-grown cells and clustered by using hierarchical clustering with Euclidean distance and average linkage. Click here for file

Additional file 16 Expression profiles of genes involved in cyclic AMP – phosphokinase A pathway (cAMP-PKA) of *S. cerevisiae*. The figure provided shows the expression trend of genes involved in cyclic AMP – phosphokinase A pathway in cells grown on glucose for 5 or 24 h or on xylose for 72 h. Click here for file

Additional file 17 Images of 2-DE gels with phosphorylated proteins. Images of 2-DE gels showing the locations of Hxk2p, Glk1p, Eno2p and Eno1p in samples from cells grown for 72 h on xylose and for 5 h on glucose and stained either with phosphoprotein specific Pro-Q Diamond or Sypro Ruby. Click here for file
