# Supplementary material for: Regulation of xylose metabolism in recombinant Saccharomyces cerevisiae
Source: Microb Cell Fact. 2008 Jun 4;7:18. doi: 10.1186/1475-2859-7-18 (PMC2435516; doi:10.1186/1475-2859-7-18)
Supplement: Additional file 1 — Pearson correlation coefficient values between the biological and technical replicate arrays from samples of glucose fermentations. The data provided shows the Pearson correlation coefficient values between the biological and technical replicate arrays of from samples of glucose fermentations (Glc5h and Glc24h). [file 1475-2859-7-18-S1.doc]

**Additional file 1.** Pearson correlation coefficient values between the biological and technical replicate arrays of glucose fermentations (Glc5h and Glc24h). H0, H1, H2 correspond to the first, second and third biological replicate and H2.1, H2.2 and H2.3 to the first, second and third technical replicate.

|  | Glc5h | | | |  | Glc24h | | | |
| --- | --- | --- | --- | --- | --- | --- | --- | --- | --- |
|  | H1 | H2.1 | H2.2 | H2.3 |  | H1 | H2.1 | H2.2 | H2.3 |
| H0 | 0.96 | 0.95 | 0.95 | 0.95 |  | 0.94 | 0.97 | 0.97 | 0.97 |
| H1 | - | 0.96 | 0.97 | 0.97 |  | - | 0.94 | 0.95 | 0.95 |
| H2.1 | - | - | 1.00 | 1.00 |  | - | - | 0.99 | 0.99 |
| H2.2 | - | - | - | 1.00 |  | - | - | - | 1.00 |
