# Supplementary material for: Regulation of xylose metabolism in recombinant Saccharomyces cerevisiae
Source: Microb Cell Fact. 2008 Jun 4;7:18. doi: 10.1186/1475-2859-7-18 (PMC2435516; doi:10.1186/1475-2859-7-18)
Supplement: Additional file 2 — Pearson correlation coefficient values between the biological and technical replicate arrays of samples from xylose fermentations. The data provided shows the Pearson correlation coefficient values between the biological and technical replicate arrays of xylose samples from fermentations (Xyl72h). [file 1475-2859-7-18-S2.doc]

**Additional file 2.** Pearson correlation coefficient values between the biological and technical replicate arrays of xylose fermentations (Xyl72h) (Notations as in Table 1).

|  | Xyl72h | | | |
| --- | --- | --- | --- | --- |
|  | H1 | H2.1 | H2.2 | H2.3 |
| H0 | 0.98 | 0.97 | 0.97 | 0.97 |
| H1 | - | 0.98 | 0.98 | 0.98 |
| H2.1 | - | - | 0.99 | 0.99 |
| H2.2 | - | - | - | 1.00 |
