# Supplementary material for: Regulation of xylose metabolism in recombinant Saccharomyces cerevisiae
Source: Microb Cell Fact. 2008 Jun 4;7:18. doi: 10.1186/1475-2859-7-18 (PMC2435516; doi:10.1186/1475-2859-7-18)

**Additional file 3.** Scatterplots of RMA pre-processed arrays from cells grown on glucose for 5 h. On y and x-axes are the expression values in a log_2_-scale. H0, H1, H2 correspond to the first, second and third biological replicate and H2.1, H2.2 and H2.3 to the first, second and third technical replicate.


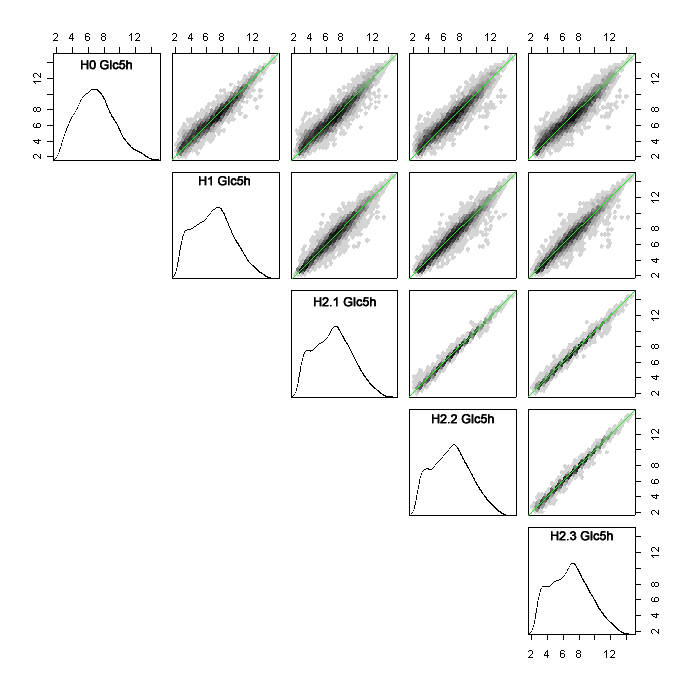

Supplement: Additional file 3 — Scatterplots of RMA pre-processed arrays from cells grown on glucose for 5 h. The figure provided represents the scatterplots of the expression values of the replicate microarrays hybridised with the samples derived from cells grown on glucose for 5 h. [file 1475-2859-7-18-S3.doc]
