# Supplementary material for: Regulation of xylose metabolism in recombinant Saccharomyces cerevisiae
Source: Microb Cell Fact. 2008 Jun 4;7:18. doi: 10.1186/1475-2859-7-18 (PMC2435516; doi:10.1186/1475-2859-7-18)
Supplement: Additional file 5 — Scatterplots of RMA pre-processed arrays from cells grown on xylose for 72 h. The figure provided represents the scatterplots of the expression values of the replicate microarrays hybridised with the samples derived from cells grown on xylose for 72 h. [file 1475-2859-7-18-S5.doc]

**Additional file 5.** Scatterplots of RMA pre-processed arrays from cells grown on xylose for 72 h. On y and x-axes are the expression values in a log_2_-scale. H0, H1, H2 correspond to the first, second and third biological replicate and H2.1, H2.2 and H2.3 to the first, second and third technical replicate.

**
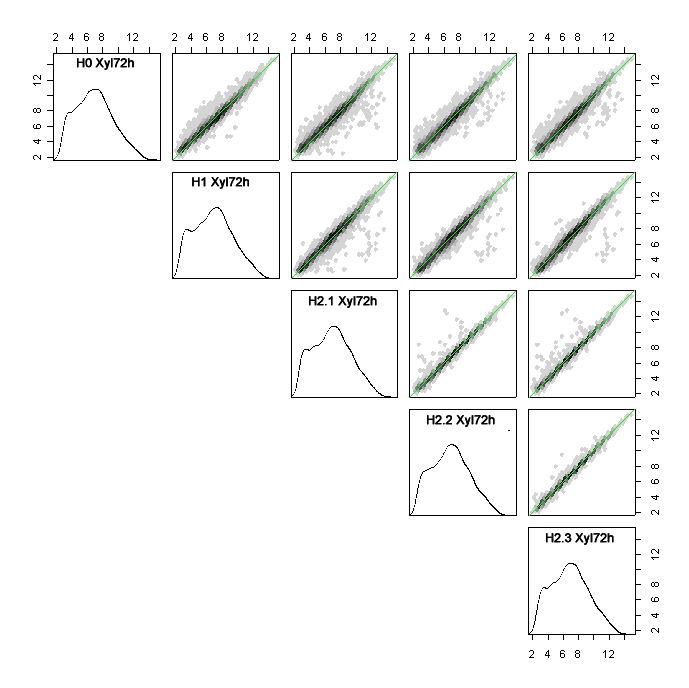
**
