# Supplementary material for: Regulation of xylose metabolism in recombinant Saccharomyces cerevisiae
Source: Microb Cell Fact. 2008 Jun 4;7:18. doi: 10.1186/1475-2859-7-18 (PMC2435516; doi:10.1186/1475-2859-7-18)
Supplement: Additional file 6 — Cluster 1. List of open reading frames in cluster 1 shown in Fig. 2 of the paper. [file 1475-2859-7-18-S6.doc]

Additional file 6.

| **ORF** | **Gene** | **Process** | **Function** |
| --- | --- | --- | --- |
| YLR409C | *UTP21* | 35S primary transcript processing | snoRNA binding |
| YJL069C | *UTP18* | 35S primary transcript processing | molecular function unknown |
| YHR065C | *RRP3* | 35S primary transcript processing | ATP-dependent RNA helicase activity |
| YNR038W | *DBP6* | 35S primary transcript processing | ATP-dependent RNA helicase activity |
| YGR158C | *MTR3* | 35S primary transcript processing | 3'-5'-exoribonuclease activity |
| YDL111C | *RRP42* | 35S primary transcript processing | 3'-5'-exoribonuclease activity |
| YGL078C | *DBP3* | 35S primary transcript processing | ATP-dependent RNA helicase activity |
| YLR186W | *EMG1* | 35S primary transcript processing | snoRNA binding |
| YHR197W | *RIX1* | 35S primary transcript processing | molecular function unknown |
| YER149C | *PEA2* | actin filament organization | cytoskeletal regulatory protein binding |
| YLR429W | *CRN1* | actin filament organization | protein binding, bridging |
| YIL138C | *TPM2* | actin filament organization | actin lateral binding |
| YMR318C | *ADH6* | aldehyde metabolism | alcohol dehydrogenase (NADP+) activity |
| YML022W | *APT1* | AMP biosynthesis | adenine phosphoribosyltransferase activity |
| YBR085W | *AAC3* | anaerobic respiration | ATP:ADP antiporter activity |
| YDR035W | *ARO3* | aromatic amino acid family biosynthesis | 3-deoxy-7-phosphoheptulonate synthase activity |
| YGL148W | *ARO2* | aromatic amino acid family biosynthesis | chorismate synthase activity |
| YGL202W | *ARO8* | aromatic amino acid family metabolism | aromatic-amino-acid transaminase activity |
| YDR321W | *ASP1* | asparagine catabolism | asparaginase activity |
| YHR019C | *DED81* | asparaginyl-tRNA aminoacylation | ATP binding |
| YDR331W | *GPI8* | attachment of GPI anchor to protein | GPI-anchor transamidase activity |
| YLR351C | *NIT3* | biological process unknown | hydrolase activity, acting on carbon-nitrogen (but not peptide) bonds |
| YDR066C |  | biological process unknown | molecular function unknown |
| YNL040W |  | biological process unknown | molecular function unknown |
| YMR122W-A |  | biological process unknown | molecular function unknown |
| YLR050C |  | biological process unknown | molecular function unknown |
| YJR124C |  | biological process unknown | molecular function unknown |
| YJL126W | *NIT2* | biological process unknown | hydrolase activity, acting on carbon-nitrogen (but not peptide) bonds |
| YAL036C | *RBG1* | biological process unknown | GTP binding |
| YHR207C | *SET5* | biological process unknown | molecular function unknown |
| YHR045W |  | biological process unknown | molecular function unknown |
| YGR026W |  | biological process unknown | molecular function unknown |
| YGL221C | *NIF3* | biological process unknown | molecular function unknown |
| YFR044C |  | biological process unknown | molecular function unknown |
| YER060W | *FCY21* | biological process unknown | cytosine-purine permease activity |
| YDL167C | *NRP1* | biological process unknown | molecular function unknown |
| YCR072C |  | biological process unknown | molecular function unknown |
| YBR227C | *MCX1* | biological process unknown | unfolded protein binding |
| YBR025C |  | biological process unknown | molecular function unknown |
| YPL226W | *NEW1* | biological process unknown | ATPase activity |
| YPL246C | *RBD2* | biological process unknown | molecular function unknown |
| YOR012W |  | biological process unknown | molecular function unknown |
| YOR012W |  | biological process unknown | molecular function unknown |
| YMR184W |  | biological process unknown | molecular function unknown |
| YML036W | *CGI121* | biological process unknown | molecular function unknown |
| YLR437C |  | biological process unknown | molecular function unknown |
| YLR243W |  | biological process unknown | signal sequence binding |
| YLR104W |  | biological process unknown | molecular function unknown |
| YLR108C |  | biological process unknown | molecular function unknown |
| YKL069W |  | biological process unknown | molecular function unknown |
| YCR087C-A |  | biological process unknown | molecular function unknown |
| YBR261C |  | biological process unknown | S-adenosylmethionine-dependent methyltransferase activity |
| YBR162C | *TOS1* | biological process unknown | molecular function unknown |
| YPL183C |  | biological process unknown | molecular function unknown |
| YOR271C |  | biological process unknown | molecular function unknown |
| YOL092W |  | biological process unknown | molecular function unknown |
| YOL154W | *ZPS1* | biological process unknown | molecular function unknown |
| YJL200C |  | biological process unknown | aconitate hydratase activity |
| YAR073W | *IMD1* | biological process unknown | molecular function unknown |
| YAR075W |  | biological process unknown | molecular function unknown |
| YGR283C |  | biological process unknown | molecular function unknown |
| YGR187C | *HGH1* | biological process unknown | molecular function unknown |
| YDR248C |  | biological process unknown | molecular function unknown |
| YOL019W |  | biological process unknown | molecular function unknown |
| YLR065C |  | biological process unknown | molecular function unknown |
| YKL128C | *PMU1* | biological process unknown | molecular function unknown |
| YHR029C | *YHI9* | biological process unknown | molecular function unknown |
| YER007C-A | *RBF20* | biological process unknown | RNA binding |
| YDR034W-B |  | biological process unknown | molecular function unknown |
| YCR015C |  | biological process unknown | molecular function unknown |
| YER156C |  | biological process unknown | molecular function unknown |
| YOR238W |  | biological process unknown | molecular function unknown |
| YDR221W |  | biological process unknown | molecular function unknown |
| YGL258W |  | biological process unknown | molecular function unknown |
| YPL245W |  | biological process unknown | molecular function unknown |
| YMR215W | *GAS3* | biological process unknown | 1,3-beta-glucanosyltransferase activity |
| YKR043C |  | biological process unknown | molecular function unknown |
| YKL056C | *RBF18* | biological process unknown | molecular function unknown |
| YIL158W |  | biological process unknown | molecular function unknown |
| YGL157W |  | biological process unknown | oxidoreductase activity |
| YGL258W |  | biological process unknown | molecular function unknown |
| YER049W | *TPA1* | biological process unknown | molecular function unknown |
| YEL001C |  | biological process unknown | molecular function unknown |
| YDR210W |  | biological process unknown | molecular function unknown |
| YDL241W |  | biological process unknown | molecular function unknown |
| YBR238C |  | biological process unknown | molecular function unknown |
| YOR387C |  | biological process unknown | molecular function unknown |
| YOR315W |  | biological process unknown | molecular function unknown |
| YOR051C |  | biological process unknown | molecular function unknown |
| YOL014W |  | biological process unknown | molecular function unknown |
| YNL175C | *NOP13* | biological process unknown | RNA binding |
| YMR116C | *ASC1* | biological process unknown | molecular function unknown |
| YMR116C | *ASC1* | biological process unknown | molecular function unknown |
| YML056C | *IMD4* | biological process unknown | IMP dehydrogenase activity |
| YML056C | *IMD4* | biological process unknown | IMP dehydrogenase activity |
| YBL083C |  | biological process unknown | molecular function unknown |
| YNL114C |  | biological process unknown | molecular function unknown |
| YLR169W |  | biological process unknown | molecular function unknown |
| YOR013W |  | biological process unknown | molecular function unknown |
| YGR151C |  | biological process unknown | molecular function unknown |
| YOR309C |  | biological process unknown | molecular function unknown |
| YOR309C |  | biological process unknown | molecular function unknown |
| YOR280C | *FSH3* | biological process unknown | serine hydrolase activity |
| YGR152C | *RSR1* | bipolar bud site selection | GTPase activity |
| YPR113W | *PIS1* | cell cycle | CDP-diacylglycerol-inositol 3-phosphatidyltransferase activity |
| YJL158C | *CIS3* | cell wall organization and biogenesis | structural constituent of cell wall |
| YEL040W | *UTR2* | cell wall organization and biogenesis | molecular function unknown |
| YLR300W | *EXG1* | cell wall organization and biogenesis | glucan 1,3-beta-glucosidase activity |
| YGR229C | *SMI1* | cell wall organization and biogenesis | molecular function unknown |
| YFL035C | *MOB2* | cellular morphogenesis during vegetative growth | protein kinase activator |
| YCR052W | *RSC6* | chromatin remodeling | molecular function unknown |
| YDR440W | *DOT1* | chromatin silencing at telomere | protein-lysine N-methyltransferase activity |
| YDR318W | *MCM21* | chromosome segregation | protein binding |
| YJL080C | *SCP160* | chromosome segregation | RNA binding |
| YMR305C | *SCW10* | conjugation with cellular fusion | glucosidase activity |
| YBR283C | *SSH1* | cotranslational protein targeting to  membrane | protein transporter activity |
| YLR301W |  | cotranslational protein targeting to membrane | molecular function unknown |
| YBR143C | *SUP45* | cytokinesis | translation release factor activity, codon specific |
| YGL234W | *ADE5,7* | 'de novo' IMP biosynthesis | phosphoribosylamine-glycine ligase activity |
| YML106W | *URA5* | 'de novo' pyrimidine base biosynthesis | orotate phosphoribosyltransferase activity |
| YJR069C | *HAM1* | DNA repair | molecular function unknown |
| YKL045W | *PRI2* | DNA replication initiation | alpha DNA polymerase activity |
| YOR074C | *CDC21* | DNA-dependent DNA replication | thymidylate synthase activity |
| YNL065W | *AQR1* | drug transport | monocarboxylic acid transporter activity |
| YEL027W | *CUP5* | endocytosis | hydrogen ion transporter activity |
| YCR053W | *THR4* | endocytosis | threonine synthase activity |
| YGL106W | *MLC1* | endocytosis | microfilament motor activity |
| YAL042W | *ERV46* | ER to Golgi transport | molecular function unknown |
| YML077W | *BET5* | ER to Golgi transport | molecular function unknown |
| YAR002C-A | *ERP1* | ER to Golgi transport | molecular function unknown |
| YIL044C | *AGE2* | ER to Golgi transport | ARF GTPase activator activity |
| YDL145C | *COP1* | ER to Golgi transport | molecular function unknown |
| YDL226C | *GCS1* | ER to Golgi transport | actin binding |
| YNR026C | *SEC12* | ER to Golgi transport | guanyl-nucleotide exchange factor activity |
| YDL192W | *ARF1* | ER to Golgi transport | GTPase activity |
| YIL076W | *SEC28* | ER to Golgi transport | molecular function unknown |
| YDL212W | *SHR3* | ER to Golgi transport | unfolded protein binding |
| YLR100W | *ERG27* | ergosterol biosynthesis | 3-keto sterol reductase activity |
| YHR072W | *ERG7* | ergosterol biosynthesis | lanosterol synthase activity |
| YGR175C | *ERG1* | ergosterol biosynthesis | squalene monooxygenase activity |
| YMR202W | *ERG2* | ergosterol biosynthesis | C-8 sterol isomerase activity |
| YML008C | *ERG6* | ergosterol biosynthesis | sterol 24-C-methyltransferase activity |
| YGL001C | *ERG26* | ergosterol biosynthesis | C-3 sterol dehydrogenase activity |
| YNR043W | *MVD1* | ergosterol biosynthesis | diphosphomevalonate decarboxylase activity |
| YJL196C | *ELO1* | fatty acid elongation, unsaturated fatty acid | fatty acid elongase activity |
| YGL256W | *ADH4* | fermentation | alcohol dehydrogenase activity, zinc-dependent |
| YBR171W | *SEC66* | filamentous growth | protein transporter activity |
| YMR113W | *FOL3* | folic acid and derivative biosynthesis | dihydrofolate synthase activity |
| YGL245W | *GUS1* | glutamyl-tRNA aminoacylation | glutamate-tRNA ligase activity |
| YBR196C | *PGI1* | glycolysis | glucose-6-phosphate isomerase activity |
| YHR174W | *ENO2* | glycolysis | phosphopyruvate hydratase activity |
| YBR121C | *GRS1* | glycyl-tRNA aminoacylation | glycine-tRNA ligase activity |
| YDR454C | *GUK1* | GMP metabolism | guanylate kinase activity |
| YMR217W | *GUA1* | GMP metabolism | GMP synthase (glutamine-hydrolyzing) activity |
| YHR216W | *IMD2* | GTP biosynthesis | IMP dehydrogenase activity |
| YER014W | *HEM14* | heme biosynthesis | protoporphyrinogen oxidase activity |
| YDR345C | *HXT3* | hexose transport | glucose transporter activity |
| YMR011W | *HXT2* | hexose transport | glucose transporter activity |
| YOR202W | *HIS3* | histidine biosynthesis | imidazoleglycerol-phosphate dehydratase activity |
| YIL116W | *HIS5* | histidine biosynthesis | histidinol-phosphate transaminase activity |
| YCL030C | *HIS4* | histidine biosynthesis | phosphoribosyl-ATP diphosphatase activity |
| YER055C | *HIS1* | histidine biosynthesis | ATP phosphoribosyltransferase activity |
| YOL061W | *PRS5* | histidine biosynthesis | ribose phosphate diphosphokinase activity |
| YBR248C | *HIS7* | histidine biosynthesis | imidazoleglycerol-phosphate synthase activity |
| YPR033C | *HTS1* | histidyl-tRNA aminoacylation | histidine-tRNA ligase activity |
| YHR068W | *DYS1* | hypusine biosynthesis from peptidyl- lysine | transferase activity, transferring alkyl or aryl (other than methyl) groups |
| YMR038C | *CCS1* | intracellular copper ion transport | superoxide dismutase copper chaperone activity |
| YOR080W | *DIA2* | invasive growth (sensu Saccharomyces) | molecular function unknown |
| YNR060W | *FRE4* | iron-siderophore transport | ferric-chelate reductase activity |
| YNL313C |  | karyogamy | molecular function unknown |
| YPL160W | *CDC60* | leucyl-tRNA aminoacylation | leucine-tRNA ligase activity |
| YNR050C | *LYS9* | lysine biosynthesis via aminoadipic acid | saccharopine dehydrogenase (NADP+, L-glutamate-forming) activity |
| YDR037W | *KRS1* | lysyl-tRNA aminoacylation | lysine-tRNA ligase activity |
| YOR079C | *ATX2* | manganese ion homeostasis | manganese ion transporter activity |
| YOL091W | *SPO21* | meiosis | structural molecule activity |
| YOR198C | *BFR1* | meiosis | RNA binding |
| YNL108C |  | metabolism | molecular function unknown |
| YHR025W | *THR1* | methionine metabolism | homoserine kinase activity |
| YER043C | *SAH1* | methionine metabolism | adenosylhomocysteinase activity |
| YDR158W | *HOM2* | methionine metabolism | aspartate-semialdehyde dehydrogenase activity |
| YGR264C | *MES1* | methionyl-tRNA aminoacylation | methionine-tRNA ligase activity |
| YJR070C | *LIA1* | microtubule cytoskeleton organization and  biogenesis | protein binding |
| YGL020C | *MDM39* | mitochondrion organization and biogenesis | molecular function unknown |
| YGL021W | *ALK1* | mitosis | protein serine/threonine kinase activity |
| YFL037W | *TUB2* | mitotic sister chromatid segregation | structural constituent of cytoskeleton |
| YML124C | *TUB3* | mitotic sister chromatid segregation | structural constituent of cytoskeleton |
| YNL016W | *PUB1* | mRNA catabolism, nonsense-mediated  decay | nucleic acid binding |
| YNL112W | *DBP2* | mRNA catabolism, nonsense-mediated  decay | RNA helicase activity |
| YOR046C | *DBP5* | mRNA export from nucleus | RNA helicase activity |
| YDR381W | *YRA1* | mRNA export from nucleus | RNA binding |
| YEL058W | *PCM1* | N-acetylglucosamine biosynthesis | phosphoacetylglucosamine mutase activity |
| YFR047C | *BNA6* | NAD biosynthesis | nicotinate-nucleotide diphosphorylase (carboxylating) activity |
| YJR025C | *BNA1* | NAD biosynthesis | 3-hydroxyanthranilate 3,4-dioxygenase activity |
| YPL037C | *EGD1* | nascent polypeptide association | unfolded protein binding |
| YHR193C | *EGD2* | nascent polypeptide association | unfolded protein binding |
| YLL002W | *RTT109* | negative regulation of DNA transposition | molecular function unknown |
| YCL026C-A | *FRM2* | negative regulation of fatty acid metabolism | molecular function unknown |
| YOR276W | *CAF20* | negative regulation of translation | translation regulator activity |
| YGR278W | *CWC22* | nuclear mRNA splicing, via spliceosome | molecular function unknown |
| YDR235W | *PRP42* | nuclear mRNA splicing, via spliceosome | RNA binding |
| YDL084W | *SUB2* | nuclear mRNA splicing, via spliceosome | protein binding |
| YEL026W | *SNU13* | nuclear mRNA splicing, via spliceosome | RNA binding |
| YBR084W | *MIS1* | nucleobase, nucleoside, nucleotide and nucleic acid metabolism | formate-tetrahydrofolate ligase activity |
| YKR092C | *SRP40* | nucleocytoplasmic transport | unfolded protein binding |
| YCL050C | *APA1* | nucleotide metabolism | bis(5'-nucleosyl)-tetraphosphatase activity |
| YDR226W | *ADK1* | nucleotide metabolism | adenylate kinase activity |
| YIL145C | *PAN6* | pantothenate biosynthesis | pantoate-beta-alanine ligase activity |
| YHR063C | *PAN5* | pantothenate biosynthesis | 2-dehydropantoate 2-reductase activity |
| YJL121C | *RPE1* | pentose-phosphate shunt | ribulose-phosphate 3-epimerase activity |
| YPR074C | *TKL1* | pentose-phosphate shunt | transketolase activity |
| YIL103W | *DPH1* | peptidyl-diphthamide biosynthesis from peptidyl-histidine | molecular function unknown |
| YFL022C | *FRS2* | phenylalanyl-tRNA aminoacylation | phenylalanine-tRNA ligase activity |
| YLR060W | *FRS1* | phenylalanyl-tRNA aminoacylation | phenylalanine-tRNA ligase activity |
| YBR092C | *PHO3* | phosphate metabolism | acid phosphatase activity |
| YBR106W | *PHO88* | phosphate transport | phosphate transporter activity |
| YBL039C | *URA7* | phospholipid biosynthesis | CTP synthase activity |
| YHR201C | *PPX1* | polyphosphate metabolism | exopolyphosphatase activity |
| YOR281C | *PLP2* | positive regulation of transcription from RNA polymerase II promoter by pheromones | GTPase inhibitor activity |
| YLR292C | *SEC72* | posttranslational protein targeting to membrane | protein transporter activity |
| YDR398W | *UTP5* | processing of 20S pre-rRNA | snoRNA binding |
| YDR324C | *UTP4* | processing of 20S pre-rRNA | snoRNA binding |
| YML093W | *UTP14* | processing of 20S pre-rRNA | snoRNA binding |
| YLR222C | *UTP13* | processing of 20S pre-rRNA | snoRNA binding |
| YGR128C | *UTP8* | processing of 20S pre-rRNA | snoRNA binding |
| YER023W | *PRO3* | proline biosynthesis | pyrroline-5-carboxylate reductase activity |
| YOR253W | *NAT5* | protein amino acid acetylation | peptide alpha-N-acetyltransferase activity |
| YGR123C | *PPT1* | protein amino acid dephosphorylation | protein serine/threonine phosphatase activity |
| YFR028C | *CDC14* | protein amino acid dephosphorylation | phosphoprotein phosphatase activity |
| YFR003C | *YPI1* | protein amino acid dephosphorylation | protein phosphatase inhibitor activity |
| YDL236W | *PHO13* | protein amino acid dephosphorylation | alkaline phosphatase activity |
| YEL042W | *GDA1* | protein amino acid glycosylation | guanosine-diphosphatase activity |
| YNR030W | *ALG12* | protein amino acid glycosylation | alpha-1,6-mannosyltransferase activity |
| YER003C | *PMI40* | protein amino acid glycosylation | mannose-6-phosphate isomerase activity |
| YDL055C | *PSA1* | protein amino acid glycosylation | mannose-1-phosphate guanylyltransferase activity |
| YMR149W | *SWP1* | protein amino acid N-linked glycosylation | dolichyl-diphosphooligosaccharide-protein glycotransferase activity |
| YGL022W | *STT3* | protein amino acid N-linked glycosylation | dolichyl-diphosphooligosaccharide-protein  glycotransferase activity |
| YGL226C-A | *OST5* | protein amino acid N-linked glycosylation | dolichyl-diphosphooligosaccharide-protein  glycotransferase activity |
| YDL232W | *OST4* | protein amino acid N-linked glycosylation | protein binding, bridging |
| YPL227C | *ALG5* | protein amino acid N-linked glycosylation | dolichyl-phosphate beta-glucosyltransferase  activity |
| YJL002C | *OST1* | protein amino acid N-linked glycosylation | dolichyl-diphosphooligosaccharide-protein  glycotransferase activity |
| YEL002C | *WBP1* | protein amino acid N-linked glycosylation | dolichyl-diphosphooligosaccharide-protein  glycotransferase activity |
| YGL225W | *VRG4* | protein amino acid N-linked glycosylation | nucleotide-sugar transporter activity |
| YPR183W | *DPM1* | protein amino acid N-linked glycosylation | transferase activity, transferring glycosyl groups |
| YJR143C | *PMT4* | protein amino acid O-linked glycosylation | dolichyl-phosphate-mannose-protein  mannosyltransferase activity |
| YBR160W | *CDC28* | protein amino acid phosphorylation | cyclin-dependent protein kinase activity |
| YKL166C | *TPK3* | protein amino acid phosphorylation | protein serine/threonine kinase activity |
| YGL019W | *CKB1* | protein amino acid phosphorylation | protein kinase CK2 regulator activity |
| YOR061W | *CKA2* | protein amino acid phosphorylation | protein kinase CK2 activity |
| YLL018C | *DPS1* | protein biosynthesis | RNA binding |
| YHL001W | *RPL14B* | protein biosynthesis | structural constituent of ribosome |
| YHL001W | *RPL14B* | protein biosynthesis | structural constituent of ribosome |
| YHL033C | *RPL8A* | protein biosynthesis | structural constituent of ribosome |
| YGR148C | *RPL24B* | protein biosynthesis | structural constituent of ribosome |
| YGR027C | *RPS25A* | protein biosynthesis | structural constituent of ribosome |
| YGL103W | *RPL28* | protein biosynthesis | structural constituent of ribosome |
| YGL189C | *RPS26A* | protein biosynthesis | structural constituent of ribosome |
| YFR031C-A | *RPL2A* | protein biosynthesis | structural constituent of ribosome |
| YDR500C | *RPL37B* | protein biosynthesis | structural constituent of ribosome |
| YDL075W | *RPL31A* | protein biosynthesis | structural constituent of ribosome |
| YDL075W | *RPL31A* | protein biosynthesis | structural constituent of ribosome |
| YBL072C | *RPS8A* | protein biosynthesis | structural constituent of ribosome |
| YPL079W | *RPL21B* | protein biosynthesis | structural constituent of ribosome |
| YNL209W | *SSB2* | protein biosynthesis | ATPase activity |
| YMR230W | *RPS10B* | protein biosynthesis | structural constituent of ribosome |
| YMR194W | *RPL36A* | protein biosynthesis | structural constituent of ribosome |
| YLR333C | *RPS25B* | protein biosynthesis | structural constituent of ribosome |
| YKR057W | *RPS21A* | protein biosynthesis | structural constituent of ribosome |
| YJL177W | *RPL17B* | protein biosynthesis | structural constituent of ribosome |
| YHR010W | *RPL27A* | protein biosynthesis | structural constituent of ribosome |
| YDR341C |  | protein biosynthesis | arginine-tRNA ligase activity |
| YPL143W | *RPL33A* | protein biosynthesis | structural constituent of ribosome |
| YPL249C-A | *RPL36B* | protein biosynthesis | structural constituent of ribosome |
| YOR369C | *RPS12* | protein biosynthesis | structural constituent of ribosome |
| YOR293W | *RPS10A* | protein biosynthesis | structural constituent of ribosome |
| YOR167C | *RPS28A* | protein biosynthesis | structural constituent of ribosome |
| YOR096W | *RPS7A* | protein biosynthesis | structural constituent of ribosome |
| YLR441C | *RPS1A* | protein biosynthesis | structural constituent of ribosome |
| YLL045C | *RPL8B* | protein biosynthesis | structural constituent of ribosome |
| YGR027C | *RPS25A* | protein biosynthesis | structural constituent of ribosome |
| YFR032C-A | *RPL29* | protein biosynthesis | structural constituent of ribosome |
| YER074W | *RPS24A* | protein biosynthesis | structural constituent of ribosome |
| YER056C-A | *RPL34A* | protein biosynthesis | structural constituent of ribosome |
| YDR064W | *RPS13* | protein biosynthesis | structural constituent of ribosome |
| YLL045C | *RPL8B* | protein biosynthesis | structural constituent of ribosome |
| YHL015W | *RPS20* | protein biosynthesis | structural constituent of ribosome |
| YBL076C | *ILS1* | protein biosynthesis | isoleucine-tRNA ligase activity |
| YJR094W-A | *RPL43B* | protein biosynthesis | structural constituent of ribosome |
| YGL076C | *RPL7A* | protein biosynthesis | structural constituent of ribosome |
| YLR061W | *RPL22A* | protein biosynthesis | structural constituent of ribosome |
| YJL177W | *RPL17B* | protein biosynthesis | structural constituent of ribosome |
| YJL190C | *RPS22A* | protein biosynthesis | structural constituent of ribosome |
| YIL078W | *THS1* | protein biosynthesis | threonine-tRNA ligase activity |
| YHR064C | *SSZ1* | protein biosynthesis | unfolded protein binding |
| YGL076C | *RPL7A* | protein biosynthesis | structural constituent of ribosome |
| YER131W | *RPS26B* | protein biosynthesis | structural constituent of ribosome |
| YDL082W | *RPL13A* | protein biosynthesis | structural constituent of ribosome |
| YOR167C | *RPS28A* | protein biosynthesis | structural constituent of ribosome |
| YOR096W | *RPS7A* | protein biosynthesis | structural constituent of ribosome |
| YLR367W | *RPS22B* | protein biosynthesis | structural constituent of ribosome |
| YLR367W | *RPS22B* | protein biosynthesis | structural constituent of ribosome |
| YIL052C | *RPL34B* | protein biosynthesis | structural constituent of ribosome |
| YGR214W | *RPS0A* | protein biosynthesis | structural constituent of ribosome |
| YGL030W | *RPL30* | protein biosynthesis | structural constituent of ribosome |
| YGL123W | *RPS2* | protein biosynthesis | structural constituent of ribosome |
| YCR031C | *RPS14A* | protein biosynthesis | structural constituent of ribosome |
| YBR191W | *RPL21A* | protein biosynthesis | structural constituent of ribosome |
| YLR048W | *RPS0B* | protein biosynthesis | structural constituent of ribosome |
| YDR418W | *RPL12B* | protein biosynthesis | structural constituent of ribosome |
| YBR061C | *TRM7* | protein biosynthesis | tRNA methyltransferase activity |
| YPR102C | *RPL11A* | protein biosynthesis | structural constituent of ribosome |
| YBR189W | *RPS9B* | protein biosynthesis | structural constituent of ribosome |
| YLR448W | *RPL6B* | protein biosynthesis | structural constituent of ribosome |
| YBR189W | *RPS9B* | protein biosynthesis | structural constituent of ribosome |
| YOL026C | *MIM1* | protein complex assembly | molecular function unknown |
| YHR060W | *VMA22* | protein complex assembly | unfolded protein binding |
| YDR518W | *EUG1* | protein folding | protein disulfide isomerase activity |
| YLL024C | *SSA2* | protein folding | ATP binding |
| YAL058W | *CNE1* | protein folding | unfolded protein binding |
| YKL084W | *HOT13* | protein import into mitochondrial  intermembrane space | molecular function unknown |
| YER009W | *NTF2* | protein import into nucleus | Ran GTPase binding |
| YMR274C | *RCE1* | protein processing | prenyl-dependent CAAX protease activity |
| YFL045C | *SEC53* | protein targeting to ER | phosphomannomutase activity |
| YKL122C | *SRP21* | protein targeting to ER | signal sequence binding |
| YNL246W | *VPS75* | protein targeting to vacuole | molecular function unknown |
| YOL098C |  | proteolysis | metalloendopeptidase activity |
| YDR144C | *MKC7* | proteolysis | aspartic-type signal peptidase activity |
| YJR105W | *ADO1* | purine base metabolism | adenosine kinase activity |
| YGL224C | *SDT1* | pyrimidine base metabolism | nucleotidase activity |
| YBR252W | *DUT1* | pyrimidine deoxyribonucleoside  triphosphate catabolism | dUTP diphosphatase activity |
| YHR144C | *DCD1* | pyrimidine nucleotide metabolism | dCMP deaminase activity |
| YHR128W | *FUR1* | pyrimidine salvage | uracil phosphoribosyltransferase activity |
| YDR400W | *URH1* | pyrimidine salvage | uridine nucleosidase activity |
| YDR353W | *TRR1* | regulation of cell redox homeostasis | thioredoxin-disulfide reductase activity |
| YGR211W | *ZPR1* | regulation of progression through cell cycle | protein binding |
| YML127W | *RSC9* | regulation of transcription from RNA  polymerase II promoter | chromatin binding |
| YDR190C | *RVB1* | regulation of transcription from RNA  polymerase II promoter | ATPase activity |
| YGL209W | *MIG2* | regulation of transcription from RNA  polymerase II promoter | specific RNA polymerase II transcription factor activity |
| YPR052C | *NHP6A* | regulation of transcription from RNA  polymerase II promoter | chromatin binding |
| YPL038W | *MET31* | regulation of transcription | DNA binding |
| YGL253W | *HXK2* | replicative cell aging | hexokinase activity |
| YFL010C | *WWM1* | response to desiccation | molecular function unknown |
| YNL264C | *PDR17* | response to drug | phosphatidylinositol transporter activity |
| YDR162C | *NBP2* | response to heat | molecular function unknown |
| YDL235C | *YPD1* | response to osmotic stress | transferase activity, transferring phosphorus- containing groups |
| YIL053W | *RHR2* | response to osmotic stress | glycerol-1-phosphatase activity |
| YHR183W | *GND1* | response to oxidative stress | phosphogluconate dehydrogenase  (decarboxylating) activity |
| YGR253C | *PUP2* | response to stress | endopeptidase activity |
| YDR184C | *ATC1* | response to stress | molecular function unknown |
| YBR082C | *UBC4* | response to stress | ubiquitin conjugating enzyme activity |
| YHR043C | *DOG2* | response to stress | 2-deoxyglucose-6-phosphatase activity |
| YDR060W | *MAK21* | ribosomal large subunit assembly and  maintenance | molecular function unknown |
| YIR012W | *SQT1* | ribosomal large subunit assembly and  maintenance | molecular function unknown |
| YOL077C | *BRX1* | ribosomal large subunit assembly and  maintenance | rRNA primary transcript binding |
| YKR081C | *RPF2* | ribosomal large subunit assembly and  maintenance | rRNA binding |
| YDR101C | *ARX1* | ribosomal large subunit biogenesis | molecular function unknown |
| YER126C | *NSA2* | ribosomal large subunit biogenesis | molecular function unknown |
| YOR272W | *YTM1* | ribosomal large subunit biogenesis | molecular function unknown |
| YNL135C | *FPR1* | ribosome assembly | peptidyl-prolyl cis-trans isomerase activity |
| YMR131C | *RRB1* | ribosome biogenesis | molecular function unknown |
| YPL093W | *NOG1* | ribosome export from nucleus | GTPase activity |
| SNR76 |  | RNA metabolism | small nucleolar RNA |
| SNR75 |  | RNA metabolism | small nucleolar RNA |
| SNR128 |  | RNA metabolism | small nucleolar RNA |
| SNR77 |  | RNA metabolism | small nucleolar RNA |
| YNL075W | *IMP4* | rRNA modification | rRNA primary transcript binding |
| YHR148W | *IMP3* | rRNA modification | snoRNA binding |
| YDL208W | *NHP2* | rRNA modification | RNA binding |
| YLR197W | *SIK1* | rRNA modification | molecular function unknown |
| YLR175W | *CBF5* | rRNA modification | pseudouridylate synthase activity |
| YHR089C | *GAR1* | rRNA modification | RNA binding |
| YDL014W | *NOP1* | rRNA modification | methyltransferase activity |
| YOR310C | *NOP58* | rRNA modification | molecular function unknown |
| YMR049C | *ERB1* | rRNA processing | molecular function unknown |
| YDR083W | *RRP8* | rRNA processing | methyltransferase activity |
| YER006W | *NUG1* | rRNA processing | GTPase activity |
| YNL061W | *NOP2* | rRNA processing | S-adenosylmethionine-dependent  methyltransferase activity |
| YMR290C | *HAS1* | rRNA processing | RNA binding |
| YLR196W | *PWP1* | rRNA processing | molecular function unknown |
| YBR247C | *ENP1* | rRNA processing | snoRNA binding |
| YBR142W | *MAK5* | rRNA processing | ATP-dependent RNA helicase activity |
| YHR062C | *RPP1* | rRNA processing | ribonuclease P activity |
| YCL059C | *KRR1* | rRNA processing | molecular function unknown |
| YKL009W | *MRT4* | rRNA processing | molecular function unknown |
| YGR208W | *SER2* | serine family amino acid biosynthesis | phosphoserine phosphatase activity |
| YDR023W | *SES1* | seryl-tRNA aminoacylation | serine-tRNA ligase activity |
| YIR022W | *SEC11* | signal peptide processing | signal peptidase activity |
| YLR066W | *SPC3* | signal peptide processing | signal peptidase activity |
| YFL047W | *RGD2* | small GTPase mediated signal  transduction | Rho GTPase activator activity |
| YIL104C | *SHQ1* | snoRNA metabolism | molecular function unknown |
| YLR372W | *SUR4* | sphingolipid biosynthesis | fatty acid elongase activity |
| YML052W | *SUR7* | sporulation (sensu Fungi) | molecular function unknown |
| YIR026C | *YVH1* | sporulation (sensu Fungi) | protein tyrosine phosphatase activity |
| YMR079W | *SEC14* | sporulation (sensu Fungi) | phosphatidylinositol transporter activity |
| YNL111C | *CYB5* | sterol biosynthesis | electron transporter activity |
| YOR341W | *RPA190* | transcription from RNA polymerase I  promoter | DNA-directed RNA polymerase activity |
| YOR340C | *RPA43* | transcription from RNA polymerase I  promoter | DNA-directed RNA polymerase activity |
| YJR063W | *RPA12* | transcription from RNA polymerase I  promoter | DNA-directed RNA polymerase activity |
| YJL148W | *RPA34* | transcription from RNA polymerase I  promoter | DNA-directed RNA polymerase activity |
| YNL248C | *RPA49* | transcription from RNA polymerase I  promoter | DNA-directed RNA polymerase activity |
| YPR110C | *RPC40* | transcription from RNA polymerase I  promoter | DNA-directed RNA polymerase activity |
| YNL113W | *RPC19* | transcription from RNA polymerase I  promoter | DNA-directed RNA polymerase activity |
| YOR210W | *RPB10* | transcription from RNA polymerase II  promoter | DNA-directed RNA polymerase activity |
| YPR187W | *RPO26* | transcription from RNA polymerase II  promoter | DNA-directed RNA polymerase activity |
| YBR154C | *RPB5* | transcription from RNA polymerase II  promoter | DNA-directed RNA polymerase activity |
| YOR224C | *RPB8* | transcription from RNA polymerase II  promoter | DNA-directed RNA polymerase activity |
| YDL219W | *DTD1* | translation | hydrolase activity, acting on ester bonds |
| YOR133W | *EFT1* | translational elongation | translation elongation factor activity |
| YLR249W | *YEF3* | translational elongation | translation elongation factor activity |
| YAL003W | *EFB1* | translational elongation | translation elongation factor activity |
| YKL081W | *TEF4* | translational elongation | translation elongation factor activity |
| YKL081W | *TEF4* | translational elongation | translation elongation factor activity |
| YDR382W | *RPP2B* | translational elongation | structural constituent of ribosome |
| YDL081C | *RPP1A* | translational elongation | structural constituent of ribosome |
| YDL130W | *RPP1B* | translational elongation | structural constituent of ribosome |
| YDL130W | *RPP1B* | translational elongation | structural constituent of ribosome |
| YOL039W | *RPP2A* | translational elongation | structural constituent of ribosome |
| YMR146C | *TIF34* | translational initiation | translation initiation factor activity |
| YJR007W | *SUI2* | translational initiation | translation initiation factor activity |
| YDR429C | *TIF35* | translational initiation | translation initiation factor activity |
| YPL237W | *SUI3* | translational initiation | translation initiation factor activity |
| YPR163C | *TIF3* | translational initiation | translation initiation factor activity |
| YKR026C | *GCN3* | translational initiation | translation initiation factor activity |
| YJR047C | *ANB1* | translational initiation | translation initiation factor activity |
| YOR361C | *PRT1* | translational initiation | translation initiation factor activity |
| YMR260C | *TIF11* | translational initiation | translation initiation factor activity |
| YNL062C | *GCD10* | translational initiation | tRNA binding |
| YOL139C | *CDC33* | translational initiation | translation initiation factor activity |
| YDR091C | *RLI1* | translational initiation | ATPase activity |
| YDL119C |  | transport | transporter activity |
| YLR083C | *EMP70* | transport | transporter activity |
| YBR104W | *YMC2* | transport | transporter activity |
| YHR020W |  | tRNA aminoacylation for protein  translation | proline-tRNA ligase activity |
| YGL105W | *ARC1* | tRNA export from nucleus | tRNA binding |
| YDR165W | *TRM82* | tRNA methylation | protein binding |
| YOL093W | *TRM10* | tRNA methylation | tRNA (guanine) methyltransferase activity |
| YPL212C | *PUS1* | tRNA modification | tRNA-pseudouridine synthase activity |
| YOR274W | *MOD5* | tRNA modification | tRNA isopentenyltransferase activity |
| YGL232W | *TAN1* | tRNA modification | RNA binding |
| YOR243C | *PUS7* | tRNA modification | pseudouridine synthase activity |
| YDL051W | *LHP1* | tRNA processing | RNA binding |
| YHR163W | *SOL3* | tRNA processing | 6-phosphogluconolactonase activity |
| YGL026C | *TRP5* | tryptophan biosynthesis | tryptophan synthase activity |
| YER090W | *TRP2* | tryptophan biosynthesis | anthranilate synthase activity |
| YOL097C | *WRS1* | tryptophanyl-tRNA aminoacylation | tryptophan-tRNA ligase activity |
| YGR078C | *PAC10* | tubulin folding | tubulin binding |
| YGR185C | *TYS1* | tyrosyl-tRNA aminoacylation | tyrosine-tRNA ligase activity |
| YGL120C | *PRP43* | U2-type spliceosome disassembly | RNA splicing factor activity, transesterification  mechanism |
| YDR002W | *YRB1* | ubiquitin-dependent protein catabolism | Ran GTPase binding |
| YBR127C | *VMA2* | vacuolar acidification | hydrogen-transporting ATPase activity,  rotational mechanism |
| YPR036W | *VMA13* | vacuolar acidification | hydrogen-transporting ATPase activity,  rotational mechanism |
| YHR026W | *PPA1* | vacuolar acidification | hydrogen-transporting ATPase activity,  rotational mechanism |
| YKL080W | *VMA5* | vacuolar acidification | hydrogen-transporting ATPase activity, rotational mechanism |
| YGR020C | *VMA7* | vacuolar acidification | hydrogen-transporting ATPase activity,  rotational mechanism |
| YGR094W | *VAS1* | valyl-tRNA aminoacylation | valine-tRNA ligase activity |
| YDL015C | *TSC13* | very-long-chain fatty acid metabolism | oxidoreductase activity |
| YMR183C | *SSO2* | vesicle fusion | t-SNARE activity |
| YAR033W | *MST28* | vesicle organization and biogenesis | protein binding |
| YCR034W | *FEN1* | vesicle-mediated transport | fatty acid elongase activity |
