# Supplementary material for: Regulation of xylose metabolism in recombinant Saccharomyces cerevisiae
Source: Microb Cell Fact. 2008 Jun 4;7:18. doi: 10.1186/1475-2859-7-18 (PMC2435516; doi:10.1186/1475-2859-7-18)
Supplement: Additional file 7 — Cluster 2. List of open reading frames in cluster 2 shown in Fig. 2 of the paper. [file 1475-2859-7-18-S7.doc]

### Additional file 7.

| **ORF** | Gene | **Process** | **Function** |
| --- | --- | --- | --- |
| YLR430W | *SEN1* | 35S primary transcript processing | ATP-dependent RNA helicase activity |
| YBL015W | *ACH1* | acetate metabolism | acetyl-CoA hydrolase activity |
| YAL054C | *ACS1* | acetyl-CoA biosynthesis | acetate-CoA ligase activity |
| YLR305C | *STT4* | actin cytoskeleton organization and  biogenesis | 1-phosphatidylinositol 4-kinase activity |
| YNL094W | *APP1* | actin filament organization | molecular function unknown |
| YBL045C | *COR1* | aerobic respiration | ubiquinol-cytochrome-c reductase activity |
| YPL270W | *MDL2* | aerobic respiration | ATPase activity, coupled to transmembrane  movement of substances |
| YDR529C | *QCR7* | aerobic respiration | ubiquinol-cytochrome-c reductase activity |
| YBL030C | *PET9* | aerobic respiration | ATP:ADP antiporter activity |
| YMR256C | *COX7* | aerobic respiration | cytochrome-c oxidase activity |
| YGR183C | *QCR9* | aerobic respiration | ubiquinol-cytochrome-c reductase activity |
| YGL187C | *COX4* | aerobic respiration | cytochrome-c oxidase activity |
| YEL024W | *RIP1* | aerobic respiration | ubiquinol-cytochrome-c reductase activity |
| YNL052W | *COX5A* | aerobic respiration | cytochrome-c oxidase activity |
| YDL174C | *DLD1* | aerobic respiration | D-lactate dehydrogenase (cytochrome) activity |
| YMR056C | *AAC1* | aerobic respiration | ATP:ADP antiporter activity |
| YBR026C | *ETR1* | aerobic respiration | enoyl-[acyl-carrier protein] reductase activity |
| YPR155C | *NCA2* | aerobic respiration | molecular function unknown |
| YHR011W | *DIA4* | aerobic respiration | serine-tRNA ligase activity |
| YML129C | *COX14* | aerobic respiration | molecular function unknown |
| YPR191W | *QCR2* | aerobic respiration | ubiquinol-cytochrome-c reductase activity |
| YAR035W | *YAT1* | alcohol metabolism | carnitine O-acetyltransferase activity |
| YER024W | *YAT2* | alcohol metabolism | carnitine O-acetyltransferase activity |
| YJR152W | *DAL5* | allantoate transport | allantoate transporter activity |
| YER119C | *AVT6* | amino acid transport | amino acid transporter activity |
| YCL025C | *AGP1* | amino acid transport | amino acid transporter activity |
| YFL055W | *AGP3* | amino acid transport | amino acid transporter activity |
| YGR121C | *MEP1* | ammonium transport | ammonium transporter activity |
| YJR096W |  | arabinose catabolism | aldo-keto reductase activity |
| YOR303W | *CPA1* | arginine biosynthesis | carbamoyl-phosphate synthase  (glutamine-hydrolyzing) activity |
| YDR173C | *ARG82* | arginine metabolism | inositol or phosphatidylinositol kinase activity |
| YDL004W | *ATP16* | ATP synthesis coupled proton transport | hydrogen-transporting ATP synthase activity,  rotational mechanism |
| YPL078C | *ATP4* | ATP synthesis coupled proton transport | structural molecule activity |
| YDR298C | *ATP5* | ATP synthesis coupled proton transport | structural molecule activity |
| YBR039W | *ATP3* | ATP synthesis coupled proton transport | hydrogen-transporting ATP synthase activity,  rotational mechanism |
| YDL181W | *INH1* | ATP synthesis coupled proton transport | enzyme inhibitor activity |
| YGR008C | *STF2* | ATP synthesis coupled proton transport | molecular function unknown |
| YBR128C | *ATG14* | autophagy | molecular function unknown |
| YGL180W | *ATG1* | autophagy | protein serine/threonine kinase activity |
| YKL187C |  | biological process unknown | molecular function unknown |
| YIL057C |  | biological process unknown | molecular function unknown |
| YGR067C |  | biological process unknown | molecular function unknown |
| YPL201C | *YIG1* | biological process unknown | molecular function unknown |
| YJL151C | *SNA3* | biological process unknown | molecular function unknown |
| YJL223C | *PAU1* | biological process unknown | molecular function unknown |
| YIL092W |  | biological process unknown | molecular function unknown |
| YML002W |  | biological process unknown | molecular function unknown |
| YER066W |  | biological process unknown | molecular function unknown |
| YER033C | *ZRG8* | biological process unknown | molecular function unknown |
| YDR387C |  | biological process unknown | permease activity |
| YDR338C |  | biological process unknown | molecular function unknown |
| YDR003W | *RCR2* | biological process unknown | molecular function unknown |
| YDL010W |  | biological process unknown | molecular function unknown |
| YCL057C-A |  | biological process unknown | molecular function unknown |
| YCL057C-A |  | biological process unknown | molecular function unknown |
| YBR239C |  | biological process unknown | molecular function unknown |
| YBL067C | *UBP13* | biological process unknown | ubiquitin-specific protease activity |
| YPL068C |  | biological process unknown | molecular function unknown |
| YPL272C |  | biological process unknown | molecular function unknown |
| YOR350C | *MNE1* | biological process unknown | molecular function unknown |
| YOR292C |  | biological process unknown | molecular function unknown |
| YOR220W |  | biological process unknown | molecular function unknown |
| YNL176C |  | biological process unknown | molecular function unknown |
| YLR278C |  | biological process unknown | molecular function unknown |
| YKL222C |  | biological process unknown | molecular function unknown |
| YER051W |  | biological process unknown | molecular function unknown |
| YEL025C |  | biological process unknown | molecular function unknown |
| YDR505C | *PSP1* | biological process unknown | molecular function unknown |
| YDR239C |  | biological process unknown | molecular function unknown |
| YDR111C | *ALT2* | biological process unknown | transaminase activity |
| YDR090C |  | biological process unknown | molecular function unknown |
| YPR117W |  | biological process unknown | molecular function unknown |
| YPL236C |  | biological process unknown | protein kinase activity |
| YOR052C |  | biological process unknown | molecular function unknown |
| YOL053W |  | biological process unknown | molecular function unknown |
| YOL087C |  | biological process unknown | molecular function unknown |
| YNL023C | *FAP1* | biological process unknown | transcription factor activity |
| YNL100W |  | biological process unknown | molecular function unknown |
| YMR306W | *FKS3* | biological process unknown | 1,3-beta-glucan synthase activity |
| YMR252C |  | biological process unknown | molecular function unknown |
| YMR253C |  | biological process unknown | molecular function unknown |
| YKL094W | *YJU3* | biological process unknown | serine hydrolase activity |
| YJL149W |  | biological process unknown | molecular function unknown |
| YIR039C | *YPS6* | biological process unknown | aspartic-type endopeptidase activity |
| YGR250C |  | biological process unknown | RNA binding |
| YEL020C |  | biological process unknown | molecular function unknown |
| YBR262C | *FMP51* | biological process unknown | molecular function unknown |
| YBR139W |  | biological process unknown | carboxypeptidase C activity |
| YLR352W |  | biological process unknown | molecular function unknown |
| YJR122W | *CAF17* | biological process unknown | molecular function unknown |
| YOR044W |  | biological process unknown | molecular function unknown |
| YMR171C |  | biological process unknown | molecular function unknown |
| YKL105C |  | biological process unknown | molecular function unknown |
| YBR284W |  | biological process unknown | molecular function unknown |
| YGR154C | *GTO1* | biological process unknown | molecular function unknown |
| YMR034C |  | biological process unknown | sterol transporter activity |
| YOL153C |  | biological process unknown | molecular function unknown |
| YKL224C |  | biological process unknown | molecular function unknown |
| YJL066C | *MPM1* | biological process unknown | molecular function unknown |
| YJL163C |  | biological process unknown | molecular function unknown |
| YJL185C |  | biological process unknown | molecular function unknown |
| YAL034C | *FUN19* | biological process unknown | molecular function unknown |
| YIL055C |  | biological process unknown | molecular function unknown |
| YER158C |  | biological process unknown | molecular function unknown |
| YDL169C | *UGX2* | biological process unknown | molecular function unknown |
| YBR230C |  | biological process unknown | molecular function unknown |
| YOL084W | *PHM7* | biological process unknown | molecular function unknown |
| YNL237W | *YTP1* | biological process unknown | molecular function unknown |
| YMR196W |  | biological process unknown | molecular function unknown |
| YLR267W | *BOP2* | biological process unknown | molecular function unknown |
| YLR149C |  | biological process unknown | molecular function unknown |
| YLR152C |  | biological process unknown | molecular function unknown |
| YJR008W |  | biological process unknown | molecular function unknown |
| YIR041W |  | biological process unknown | molecular function unknown |
| YGR201C |  | biological process unknown | molecular function unknown |
| YGR146C |  | biological process unknown | molecular function unknown |
| YFR017C |  | biological process unknown | molecular function unknown |
| YDR275W | *BSC2* | biological process unknown | molecular function unknown |
| YCR104W | *PAU3* | biological process unknown | molecular function unknown |
| YOR394W |  | biological process unknown | molecular function unknown |
| YOR173W | *DCS2* | biological process unknown | molecular function unknown |
| YNL194C |  | biological process unknown | molecular function unknown |
| YMR325W |  | biological process unknown | molecular function unknown |
| YML087C |  | biological process unknown | molecular function unknown |
| YAL061W |  | biological process unknown | oxidoreductase activity, acting on the CH-OH  group of donors, NAD or NADP as acceptor |
| YDL204W | *RTN2* | biological process unknown | molecular function unknown |
| YOR186W |  | biological process unknown | molecular function unknown |
| YMR181C |  | biological process unknown | molecular function unknown |
| YLR312C |  | biological process unknown | molecular function unknown |
| YPL186C | *UIP4* | biological process unknown | molecular function unknown |
| YLR297W |  | biological process unknown | molecular function unknown |
| YER067W |  | biological process unknown | molecular function unknown |
| YOR019W |  | biological process unknown | molecular function unknown |
| YBR033W | *EDS1* | biological process unknown | molecular function unknown |
| YPL109C |  | biological process unknown | molecular function unknown |
| YOR227W |  | biological process unknown | molecular function unknown |
| YML030W |  | biological process unknown | molecular function unknown |
| YLR422W |  | biological process unknown | molecular function unknown |
| YKR096W |  | biological process unknown | molecular function unknown |
| YKR017C |  | biological process unknown | molecular function unknown |
| YKL091C |  | biological process unknown | molecular function unknown |
| YKL162C |  | biological process unknown | molecular function unknown |
| YJR127C | *ZMS1* | biological process unknown | transcription factor activity |
| YJR039W |  | biological process unknown | molecular function unknown |
| YER188C-A |  | biological process unknown | molecular function unknown |
| YHR198C | *FMP22* | biological process unknown | molecular function unknown |
| YHL008C |  | biological process unknown | transporter activity |
| YGR127W |  | biological process unknown | molecular function unknown |
| YGR102C |  | biological process unknown | molecular function unknown |
| YGL056C | *SDS23* | biological process unknown | molecular function unknown |
| YDR444W |  | biological process unknown | molecular function unknown |
| YDL199C |  | biological process unknown | molecular function unknown |
| YDL183C |  | biological process unknown | molecular function unknown |
| YDL180W |  | biological process unknown | molecular function unknown |
| YBR280C |  | biological process unknown | molecular function unknown |
| YBR047W | *FMP23* | biological process unknown | molecular function unknown |
| YPR127W |  | biological process unknown | molecular function unknown |
| YOL138C |  | biological process unknown | molecular function unknown |
| YNL134C |  | biological process unknown | alcohol dehydrogenase (NADP+) activity |
| YMR085W |  | biological process unknown | molecular function unknown |
| YLR031W |  | biological process unknown | molecular function unknown |
| YDR504C | *SPG3* | biological process unknown | molecular function unknown |
| YBR270C | *BIT2* | biological process unknown | molecular function unknown |
| YOR152C |  | biological process unknown | molecular function unknown |
| YNL077W | *APJ1* | biological process unknown | unfolded protein binding |
| YNL115C |  | biological process unknown | molecular function unknown |
| YNL305C |  | biological process unknown | molecular function unknown |
| YBR269C | *FMP21* | biological process unknown | molecular function unknown |
| YLR156W |  | biological process unknown | molecular function unknown |
| YJL225C |  | biological process unknown | helicase activity |
| YIL077C |  | biological process unknown | molecular function unknown |
| YNL200C |  | biological process unknown | molecular function unknown |
| YJL016W |  | biological process unknown | molecular function unknown |
| YGR110W |  | biological process unknown | molecular function unknown |
| YPR115W |  | biological process unknown | molecular function unknown |
| YKR049C | *FMP46* | biological process unknown | molecular function unknown |
| YMR052C-A |  | biological process unknown | molecular function unknown |
| YLR317W |  | biological process unknown | molecular function unknown |
| YLR202C |  | biological process unknown | molecular function unknown |
| YJL142C |  | biological process unknown | molecular function unknown |
| YJR157W |  | biological process unknown | molecular function unknown |
| YHR130C |  | biological process unknown | molecular function unknown |
| YML089C |  | biological process unknown | molecular function unknown |
| YNL337W |  | biological process unknown | molecular function unknown |
| YJR162C |  | biological process unknown | molecular function unknown |
| YHR180W |  | biological process unknown | molecular function unknown |
| YER188W |  | biological process unknown | molecular function unknown |
| YER121W |  | biological process unknown | molecular function unknown |
| YMR320W |  | biological process unknown | molecular function unknown |
| YEL074W |  | biological process unknown | molecular function unknown |
| YLR311C |  | biological process unknown | molecular function unknown |
| YPR122W | *AXL1* | bud site selection | metalloendopeptidase activity |
| YGL006W | *PMC1* | calcium ion homeostasis | calcium-transporting ATPase activity |
| YIL155C | *GUT2* | carbohydrate metabolism | glycerol-3-phosphate dehydrogenase activity |
| YML042W | *CAT2* | carnitine metabolism | carnitine O-acetyltransferase activity |
| YBL043W | *ECM13* | cell wall organization and biogenesis | molecular function unknown |
| YIL146C | *ECM37* | cell wall organization and biogenesis | molecular function unknown |
| YKR076W | *ECM4* | cell wall organization and biogenesis | molecular function unknown |
| YDR293C | *SSD1* | cell wall organization and biogenesis | RNA binding |
| YMR176W | *ECM5* | cell wall organization and biogenesis | molecular function unknown |
| YBL101C | *ECM21* | cell wall organization and biogenesis | molecular function unknown |
| YJL083W | *TAX4* | cell wall organization and biogenesis | molecular function unknown |
| YER093C | *TSC11* | cell wall organization and biogenesis | protein binding |
| YGR032W | *GSC2* | cell wall organization and biogenesis | 1,3-beta-glucan synthase activity |
| YJL042W | *MHP1* | cell wall organization and biogenesis | structural constituent of cytoskeleton |
| YGR070W | *ROM1* | cell wall organization and biogenesis | signal transducer activity |
| YHR102W | *KIC1* | cell wall organization and biogenesis | kinase activity |
| YGR143W | *SKN1* | cell wall organization and biogenesis | glucosidase activity |
| YJL045W |  | cellular respiration | succinate dehydrogenase (ubiquinone) activity |
| YDR359C | *VID21* | chromatin modification | molecular function unknown |
| YDR073W | *SNF11* | chromatin remodeling | general RNA polymerase II transcription factor  activity |
| YGL163C | *RAD54* | chromatin remodeling | DNA-dependent ATPase activity |
| YPL138C | *SPP1* | chromatin silencing at telomere | histone lysine N-methyltransferase activity  (H3-K4 specific) |
| YIL083C |  | coenzyme A biosynthesis | phosphopantothenate-cysteine ligase activity |
| YPL156C | *PRM4* | conjugation with cellular fusion | molecular function unknown |
| YLR411W | *CTR3* | copper ion import | copper uptake transporter activity |
| YER141W | *COX15* | cytochrome c oxidase complex assembly | oxidoreductase activity, acting on NADH or  NADPH, heme protein as acceptor |
| YMR271C | *URA10* | 'de novo' pyrimidine base biosynthesis | orotate phosphoribosyltransferase activity |
| YKL032C | *IXR1* | DNA repair | DNA binding |
| YDR030C | *RAD28* | DNA repair | molecular function unknown |
| YGL150C | *INO80* | DNA repair | ATPase activity |
| YHR164C | *DNA2* | DNA repair | ATP-dependent DNA helicase activity |
| YPL167C | *REV3* | DNA repair | zeta DNA polymerase activity |
| YER169W | *RPH1* | DNA repair | specific transcriptional repressor activity |
| YML054C | *CYB2* | electron transport | L-lactate dehydrogenase (cytochrome) activity |
| YOR034C | *AKR2* | endocytosis | molecular function unknown |
| YOR329C | *SCD5* | endocytosis | protein binding |
| YDR059C | *UBC5* | endocytosis | ubiquitin conjugating enzyme activity |
| YLR450W | *HMG2* | ergosterol biosynthesis | hydroxymethylglutaryl-CoA reductase (NADPH)  activity |
| YDR078C | *SHU2* | error-free DNA repair | molecular function unknown |
| YER114C | *BOI2* | establishment of cell polarity (sensu Fungi) | phospholipid binding |
| YDL085W | *NDE2* | ethanol fermentation | NADH dehydrogenase activity |
| YKR009C | *FOX2* | fatty acid beta-oxidation | 3-hydroxyacyl-CoA dehydrogenase activity |
| YGL205W | *POX1* | fatty acid beta-oxidation | acyl-CoA oxidase activity |
| YIL160C | *POT1* | fatty acid beta-oxidation | acetyl-CoA C-acyltransferase activity |
| YOR100C | *CRC1* | fatty acid metabolism | carnitine:acyl carnitine antiporter activity |
| YOR377W | *ATF1* | fatty acid metabolism | alcohol O-acetyltransferase activity |
| YPL147W | *PXA1* | fatty acid transport | ATPase activity, coupled to transmembrane  movement of substances |
| YKL188C | *PXA2* | fatty acid transport | ATPase activity, coupled to transmembrane  movement of substances |
| YMR083W | *ADH3* | fermentation | alcohol dehydrogenase activity |
| YMR303C | *ADH2* | fermentation | alcohol dehydrogenase activity |
| YKR102W | *FLO10* | flocculation (sensu Saccharomyces) | mannose binding |
| YJR095W | *SFC1* | fumarate transport | succinate:fumarate antiporter activity |
| YFR040W | *SAP155* | G1/S transition of mitotic cell cycle | protein serine/threonine phosphatase activity |
| YGL096W | *TOS8* | G1/S-specific transcription in mitotic cell cycle | transcription factor activity |
| YKR097W | *PCK1* | gluconeogenesis | phosphoenolpyruvate carboxykinase (ATP) activity |
| YLR377C | *FBP1* | gluconeogenesis | fructose-bisphosphatase activity |
| YOL126C | *MDH2* | gluconeogenesis | L-malate dehydrogenase activity |
| YGL062W | *PYC1* | gluconeogenesis | pyruvate carboxylase activity |
| YMR105C | *PGM2* | glucose 1-phosphate utilization | phosphoglucomutase activity |
| YKL038W | *RGT1* | glucose metabolism | DNA binding |
| YAL062W | *GDH3* | glutamate biosynthesis | glutamate dehydrogenase activity |
| YLR174W | *IDP2* | glutamate biosynthesis | isocitrate dehydrogenase (NADP+) activity |
| YCR005C | *CIT2* | glutamate biosynthesis | citrate (Si)-synthase activity |
| YLR299W | *ECM38* | glutathione catabolism | protein-glutamine gamma-glutamyltransferase  activity |
| YHL032C | *GUT1* | glycerol metabolism | glycerol kinase activity |
| YCR098C | *GIT1* | glycerophosphodiester transport | glycerophosphodiester transporter activity |
| YKR058W | *GLG1* | glycogen biosynthesis | glycogenin glucosyltransferase activity |
| YJL137C | *GLG2* | glycogen biosynthesis | glycogenin glucosyltransferase activity |
| YLR258W | *GSY2* | glycogen biosynthesis | glycogen (starch) synthase activity |
| YEL011W | *GLC3* | glycogen biosynthesis | 1,4-alpha-glucan branching enzyme activity |
| YPR184W | *GDB1* | glycogen catabolism | 4-alpha-glucanotransferase activity |
| YER065C | *ICL1* | glyoxylate cycle | isocitrate lyase activity |
| YNL117W | *MLS1* | glyoxylate cycle | malate synthase activity |
| YDR343C | *HXT6* | hexose transport | glucose transporter activity |
| YOR064C | *YNG1* | histone acetylation | histone acetyltransferase activity |
| YIL112W | *HOS4* | histone deacetylation | NAD-dependent histone deacetylase activity |
| YOR370C | *MRS6* | intracellular protein transport | Rab escort protein activity |
| YLR094C | *GIS3* | intracellular signaling cascade | molecular function unknown |
| YPL135W | *ISU1* | iron ion homeostasis | protein binding |
| YFL041W | *FET5* | iron ion transport | ferroxidase activity |
| YLL027W | *ISA1* | iron ion transport | molecular function unknown |
| YHL040C | *ARN1* | iron-siderophore transport | siderophore-iron transporter activity |
| YKL217W | *JEN1* | lactate transport | lactate transporter activity |
| YDR380W | *ARO10* | leucine catabolism | pyruvate decarboxylase activity |
| YMR313C | *TGL3* | lipid metabolism | triacylglycerol lipase activity |
| YDR492W | *IZH1* | lipid metabolism | metal ion binding |
| YER015W | *FAA2* | lipid metabolism | long-chain-fatty-acid-CoA ligase activity |
| YNL073W | *MSK1* | lysyl-tRNA aminoacylation | lysine-tRNA ligase activity |
| YFL050C | *ALR2* | magnesium ion transport | di-, tri-valent inorganic cation transporter activity |
| YHL024W | *RIM4* | meiosis | RNA binding |
| YOR178C | *GAC1* | meiosis | protein phosphatase type 1 activity |
| YPL194W | *DDC1* | meiosis | molecular function unknown |
| YJL005W | *CYR1* | meiosis | adenylate cyclase activity |
| YDR273W | *DON1* | meiosis | molecular function unknown |
| YML128C | *MSC1* | meiotic recombination | molecular function unknown |
| YLR219W | *MSC3* | meiotic recombination | molecular function unknown |
| YPL164C | *MLH3* | meiotic recombination | molecular function unknown |
| YBR136W | *MEC1* | meiotic recombination | protein kinase activity |
| YGL184C | *STR3* | methionine biosynthesis | cystathionine beta-lyase activity |
| YML120C | *NDI1* | mitochondrial electron transport, NADH  to ubiquinone | oxidoreductase activity, acting on NADH or  NADPH, quinone or similar compound as acceptor |
| YPL134C | *ODC1* | mitochondrial transport | intracellular transporter activity |
| YGL055W | *OLE1* | mitochondrion inheritance | stearoyl-CoA 9-desaturase activity |
| YPR083W | *MDM36* | mitochondrion organization and biogenesis | molecular function unknown |
| YPR024W | *YME1* | mitochondrion organization and biogenesis | ATP-dependent peptidase activity |
| YGL219C | *MDM34* | mitochondrion organization and biogenesis | molecular function unknown |
| YBR179C | *FZO1* | mitochondrion organization and biogenesis | GTPase activity |
| YOL060C | *MAM3* | mitochondrion organization and biogenesis | molecular function unknown |
| YFR036W | *CDC26* | mitotic sister chromatid segregation | protein binding |
| YHR115C | *DMA1* | mitotic spindle checkpoint | molecular function unknown |
| YNL116W | *DMA2* | mitotic spindle checkpoint | molecular function unknown |
| YDL175C | *AIR2* | mRNA export from nucleus | molecular function unknown |
| YOR328W | *PDR10* | multidrug transport | ATPase activity, coupled to transmembrane  movement of substances |
| YIL017C | *VID28* | negative regulation of gluconeogenesis | molecular function unknown |
| YCL039W | *GID7* | negative regulation of gluconeogenesis | molecular function unknown |
| YGL197W | *MDS3* | negative regulation of sporulation | molecular function unknown |
| YDR223W | *CRF1* | negative regulation of transcription | transcriptional repressor activity |
| YBR050C | *REG2* | negative regulation of transcription from  RNA polymerase II promoter | protein phosphatase type 1 activity |
| YKL146W | *AVT3* | neutral amino acid transport | neutral amino acid transporter activity |
| YOR348C | *PUT4* | neutral amino acid transport | L-proline permease activity |
| YDL215C | *GDH2* | nitrogen compound metabolism | glutamate dehydrogenase activity |
| YDR150W | *NUM1* | nuclear migration, microtubule-mediated | tubulin binding |
| YOR185C | *GSP2* | nuclear organization and biogenesis | GTPase activity |
| YDR530C | *APA2* | nucleotide metabolism | bis(5'-nucleosyl)-tetraphosphatase activity |
| YMR201C | *RAD14* | nucleotide-excision repair, DNA damage  recognition | damaged DNA binding |
| YBR114W | *RAD16* | nucleotide-excision repair, DNA damage recognition | DNA-dependent ATPase activity |
| YGR258C | *RAD2* | nucleotide-excision repair, DNA incision,  3'-to lesion | single-stranded DNA specific  endodeoxyribonuclease activity |
| YJL103C |  | oxidative phosphorylation | molecular function unknown |
| YDR256C | *CTA1* | oxygen and reactive oxygen species metabolism | catalase activity |
| YDR479C | *PEX29* | peroxisome organization and biogenesis | molecular function unknown |
| YOR084W |  | peroxisome organization and biogenesis | lipase activity |
| YOR363C | *PIP2* | peroxisome organization and biogenesis | DNA binding |
| YAL051W | *OAF1* | peroxisome organization and biogenesis | DNA binding |
| YGR233C | *PHO81* | phosphate metabolism | cyclin-dependent protein kinase inhibitor activity |
| YJR077C | *MIR1* | phosphate transport | inorganic phosphate transporter activity |
| YER053C | *PIC2* | phosphate transport | inorganic phosphate transporter activity |
| YDR018C |  | phospholipid biosynthesis | acyltransferase activity |
| YKR067W | *GPT2* | phospholipid biosynthesis | glycerol-3-phosphate O-acyltransferase activity |
| YJR103W | *URA8* | phospholipid biosynthesis | CTP synthase activity |
| YOR298C-A | *MBF1* | positive regulation of transcription from  RNA polymerase II promoter | transcription coactivator activity |
| YMR280C | *CAT8* | positive regulation of transcription from  RNA polymerase II promoter | specific RNA polymerase II transcription factor  activity |
| YDR423C | *CAD1* | positive regulation of transcription from  RNA polymerase II promoter | RNA polymerase II transcription factor activity |
| YIR033W | *MGA2* | positive regulation of transcription from  RNA polymerase II promoter | transcriptional activator activity |
| YBR240C | *THI2* | positive regulation of transcription from  RNA polymerase II promoter | transcriptional activator activity |
| YDR421W | *ARO80* | positive regulation of transcription from  RNA polymerase II promoter | specific RNA polymerase II transcription factor  activity |
| YKR050W | *TRK2* | potassium ion homeostasis | potassium ion transporter activity |
| YPR002W | *PDH1* | propionate metabolism | molecular function unknown |
| YDR422C | *SIP1* | protein amino acid phosphorylation | AMP-activated protein kinase activity |
| YGL208W | *SIP2* | protein amino acid phosphorylation | AMP-activated protein kinase activity |
| YLR362W | *STE11* | protein amino acid phosphorylation | MAP kinase kinase kinase activity |
| YJL106W | *IME2* | protein amino acid phosphorylation | protein kinase activity |
| YPL031C | *PHO85* | protein amino acid phosphorylation | cyclin-dependent protein kinase activity |
| YJL164C | *TPK1* | protein amino acid phosphorylation | protein serine/threonine kinase activity |
| YPL140C | *MKK2* | protein amino acid phosphorylation | MAP kinase kinase activity |
| YMR139W | *RIM11* | protein amino acid phosphorylation | protein serine/threonine kinase activity |
| YLR248W | *RCK2* | protein amino acid phosphorylation | protein serine/threonine kinase activity |
| YPL203W | *TPK2* | protein amino acid phosphorylation | protein serine/threonine kinase activity |
| YDR490C | *PKH1* | protein amino acid phosphorylation | protein kinase activity |
| YOL100W | *PKH2* | protein amino acid phosphorylation | protein kinase activity |
| YNR036C |  | protein biosynthesis | structural constituent of ribosome |
| YJR113C | *RSM7* | protein biosynthesis | structural constituent of ribosome |
| YMR064W | *AEP1* | protein biosynthesis | molecular function unknown |
| YNR045W | *PET494* | protein biosynthesis | translation regulator activity |
| YLR439W | *MRPL4* | protein biosynthesis | structural constituent of ribosome |
| YFR049W | *YMR31* | protein biosynthesis | structural constituent of ribosome |
| YDR494W | *RSM28* | protein biosynthesis | structural constituent of ribosome |
| YML091C | *RPM2* | protein biosynthesis | ribonuclease P activity |
| YGR270W | *YTA7* | protein catabolism | ATPase activity |
| YGR174C | *CBP4* | protein complex assembly | molecular function unknown |
| YER017C | *AFG3* | protein complex assembly | ATPase activity |
| YKL016C | *ATP7* | protein complex assembly | structural molecule activity |
| YAL005C | *SSA1* | protein folding | ATPase activity |
| YAL005C | *SSA1* | protein folding | ATPase activity |
| YLR347C | *KAP95* | protein import into nucleus | protein carrier activity |
| YNR016C | *ACC1* | protein import into nucleus | acetyl-CoA carboxylase activity |
| YGR184C | *UBR1* | protein monoubiquitination | ubiquitin-protein ligase activity |
| YKL010C | *UFD4* | protein monoubiquitination | ubiquitin-protein ligase activity |
| YEL012W | *UBC8* | protein monoubiquitination | ubiquitin conjugating enzyme activity |
| YOR156C | *NFI1* | protein sumoylation | SUMO ligase activity |
| YLR246W | *ERF2* | protein targeting to membrane | protein-cysteine S-palmitoleyltransferase activity |
| YHR160C | *PEX18* | protein targeting to peroxisome | protein binding |
| YGR239C | *PEX21* | protein targeting to peroxisome | protein binding |
| YGR141W | *VPS62* | protein targeting to vacuole | molecular function unknown |
| YPR049C | *ATG11* | protein targeting to vacuole | molecular function unknown |
| YDL149W | *ATG9* | protein targeting to vacuole | molecular function unknown |
| YNR007C | *ATG3* | protein targeting to vacuole | molecular function unknown |
| YNL223W | *ATG4* | protein targeting to vacuole | microtubule binding |
| YNL242W | *ATG2* | protein targeting to vacuole | molecular function unknown |
| YDR313C | *PIB1* | protein ubiquitination | ubiquitin-protein ligase activity |
| YIL046W | *MET30* | protein ubiquitination | protein binding |
| YKL171W |  | proteolysis | protein kinase activity |
| YCR038C | *BUD5* | pseudohyphal growth | signal transducer activity |
| YKL185W | *ASH1* | pseudohyphal growth | specific transcriptional repressor activity |
| YNL142W | *MEP2* | pseudohyphal growth | ammonium transporter activity |
| YMR100W | *MUB1* | regulation of cell budding | molecular function unknown |
| YDR453C | *TSA2* | regulation of cell redox homeostasis | thioredoxin peroxidase activity |
| YLR178C | *TFS1* | regulation of proteolysis | lipid binding |
| YPL070W | *MUK1* | regulation of transcription | molecular function unknown |
| YJL089W | *SIP4* | regulation of transcription from  RNA polymerase II promoter | specific RNA polymerase II transcription  factor activity |
| YCR093W | *CDC39* | regulation of transcription from RNA  polymerase II promoter | 3'-5'-exoribonuclease activity |
| YEL009C | *GCN4* | regulation of transcription from RNA  polymerase II promoter | DNA binding |
| YDL170W | *UGA3* | regulation of transcription from RNA  polymerase II promoter | transcription factor activity |
| YPR023C | *EAF3* | regulation of transcription from RNA  polymerase II promoter | histone acetyltransferase activity |
| YER088C | *DOT6* | regulation of transcription from RNA  polymerase II promoter | molecular function unknown |
| YOR113W | *AZF1* | regulation of transcription, DNA-dependent | DNA binding |
| YML076C | *WAR1* | response to acid | transcription factor activity |
| YGR197C | *SNG1* | response to drug | molecular function unknown |
| YKL026C | *GPX1* | response to oxidative stress | glutathione peroxidase activity |
| YBR006W | *UGA2* | response to oxidative stress | succinate-semialdehyde dehydrogenase  [NAD(P)+] activity |
| YMR250W | *GAD1* | response to oxidative stress | glutamate decarboxylase activity |
| YOR324C | *FRT1* | response to stress | molecular function unknown |
| YKL062W | *MSN4* | response to stress | DNA binding |
| YLL026W | *HSP104* | response to stress | chaperone binding |
| YDR258C | *HSP78* | response to stress | ATPase activity |
| YDR017C | *KCS1* | response to stress | inositol or phosphatidylinositol kinase activity |
| YBR001C | *NTH2* | response to stress | alpha,alpha-trehalase activity |
| YPR026W | *ATH1* | response to stress | alpha,alpha-trehalase activity |
| YML100W | *TSL1* | response to stress | enzyme regulator activity |
| YDR001C | *NTH1* | response to stress | alpha,alpha-trehalase activity |
| YDR171W | *HSP42* | response to stress | unfolded protein binding |
| YOR380W | *RDR1* | response to xenobiotic stimulus | transcription factor activity |
| YPL133C | *RDS2* | response to xenobiotic stimulus | transcription factor activity |
| YBL033C | *RIB1* | riboflavin biosynthesis | cyclohydrolase activity |
| YHR087W |  | RNA metabolism | molecular function unknown |
| SNR51 |  | RNA metabolism | small nucleolar RNA |
| SNR41 |  | RNA metabolism | small nucleolar RNA |
| SNR70 |  | RNA metabolism | small nucleolar RNA |
| YMR302C | *PRP12* | rRNA processing | exonuclease activity |
| YIL047C | *SYG1* | signal transduction | molecular function unknown |
| YDL194W | *SNF3* | signal transduction | receptor activity |
| YDR436W | *PPZ2* | sodium ion homeostasis | protein serine/threonine phosphatase activity |
| YDR039C | *ENA2* | sodium ion transport | ATPase activity, coupled to transmembrane  movement of ions, phosphorylative mechanism |
| YDR096W | *GIS1* | spore wall assembly (sensu Fungi) | transcription factor activity |
| YDL239C | *ADY3* | spore wall assembly (sensu Fungi) | protein binding |
| YOL071W | *EMI5* | sporulation (sensu Fungi) | molecular function unknown |
| YDL013W | *HEX3* | sporulation (sensu Fungi) | DNA binding |
| YER046W | *SPO73* | sporulation (sensu Fungi) | molecular function unknown |
| YER020W | *GPA2* | sporulation (sensu Fungi) | GTPase activity |
| YEL060C | *PRB1* | sporulation | serine-type endopeptidase activity |
| YDL019C | *OSH2* | steroid biosynthesis | oxysterol binding |
| YLR189C | *ATG26* | sterol metabolism | sterol 3-beta-glucosyltransferase activity |
| YLR394W | *CST9* | synapsis | DNA binding |
| YMR136W | *GAT2* | transcription | transcription factor activity |
| YPR008W | *HAA1* | transcription initiation from RNA  polymerase II promoter | specific RNA polymerase II transcription factor  activity |
| YIL036W | *CST6* | transcription initiation from RNA  polymerase II promoter | specific RNA polymerase II transcription factor  activity |
| YKL109W | *HAP4* | transcription | transcriptional activator activity |
| YDR216W | *ADR1* | transcription | transcription factor activity |
| YNL014W | *HEF3* | translational elongation | ATPase activity |
| YJL102W | *MEF2* | translational elongation | translation elongation factor activity |
| YPL119C | *DBP1* | translational initiation | RNA helicase activity |
| YOL075C |  | transport | ATPase activity, coupled to transmembrane  movement of substances |
| YKR104W |  | transport | ATPase activity, coupled to transmembrane  movement of substances |
| YDR406W | *PDR15* | transport | ATPase activity, coupled to transmembrane  movement of substances |
| YOR192C |  | transport | transporter activity |
| YNL125C | *ESBP6* | transport | transporter activity |
| YDR536W | *STL1* | transport | transporter activity |
| YCR010C | *ADY2* | transport | transporter activity |
| YPL262W | *FUM1* | tricarboxylic acid cycle | fumarate hydratase activity |
| YKL085W | *MDH1* | tricarboxylic acid cycle | L-malate dehydrogenase activity |
| YIL125W | *KGD1* | tricarboxylic acid cycle | oxoglutarate dehydrogenase  (succinyl-transferring) activity |
| YLL041C | *SDH2* | tricarboxylic acid cycle | succinate dehydrogenase (ubiquinone) activity |
| YKL148C | *SDH1* | tricarboxylic acid cycle | succinate dehydrogenase (ubiquinone) activity |
| YPR001W | *CIT3* | tricarboxylic acid cycle | citrate (Si)-synthase activity |
| YNR001C | *CIT1* | tricarboxylic acid cycle | citrate (Si)-synthase activity |
| YKL141W | *SDH3* | tricarboxylic acid cycle | succinate dehydrogenase (ubiquinone) activity |
| YDR148C | *KGD2* | tricarboxylic acid cycle | dihydrolipoyllysine-residue succinyltransferase  activity |
| YDR178W | *SDH4* | tricarboxylic acid cycle | succinate dehydrogenase (ubiquinone) activity |
| YDL020C | *RPN4* | ubiquitin-dependent protein catabolism | transcriptional activator activity |
| YHL016C | *DUR3* | urea transport | urea transporter activity |
| YDR456W | *NHX1* | vacuolar acidification | monovalent inorganic cation transporter activity |
| YGL227W | *VID30* | vacuolar protein catabolism | molecular function unknown |
| YNL054W | *VAC7* | vacuole inheritance | enzyme regulator activity |
| YOR316C | *COT1* | zinc ion homeostasis | di-, tri-valent inorganic cation transporter activity |
