# Supplementary material for: Regulation of xylose metabolism in recombinant Saccharomyces cerevisiae
Source: Microb Cell Fact. 2008 Jun 4;7:18. doi: 10.1186/1475-2859-7-18 (PMC2435516; doi:10.1186/1475-2859-7-18)
Supplement: Additional file 8 — Cluster 3. List of open reading frames in cluster 3 shown in Fig. 2 of the paper. [file 1475-2859-7-18-S8.doc]

### Additional file 8.

| **ORF** | Gene | **Process** | **Function** |
| --- | --- | --- | --- |
| YOR197W | *MCA1* | apoptosis | caspase activity |
| YDR333C |  | biological process unknown | molecular function unknown |
| YDR266C |  | biological process unknown | molecular function unknown |
| YDR042C |  | biological process unknown | molecular function unknown |
| YOR264W | *DSE3* | biological process unknown | molecular function unknown |
| YOL107W |  | biological process unknown | molecular function unknown |
| YNR061C |  | biological process unknown | molecular function unknown |
| YMR210W |  | biological process unknown | molecular function unknown |
| YML072C | *TCB3* | biological process unknown | lipid binding |
| YLR455W |  | biological process unknown | molecular function unknown |
| YLR126C |  | biological process unknown | molecular function unknown |
| YLR137W |  | biological process unknown | molecular function unknown |
| YKL063C |  | biological process unknown | molecular function unknown |
| YJR015W |  | biological process unknown | molecular function unknown |
| YJL218W |  | biological process unknown | acetyltransferase activity |
| YHR179W | *OYE2* | biological process unknown | NADPH dehydrogenase activity |
| YHR127W |  | biological process unknown | molecular function unknown |
| YHL013C |  | biological process unknown | molecular function unknown |
| YGL082W |  | biological process unknown | molecular function unknown |
| YGL263W | *COS12* | biological process unknown | molecular function unknown |
| YFR006W |  | biological process unknown | X-Pro aminopeptidase activity |
| YDR257C | *SET7* | biological process unknown | molecular function unknown |
| YBR281C |  | biological process unknown | molecular function unknown |
| YBR007C | *DSF2* | biological process unknown | molecular function unknown |
| YPL030W |  | biological process unknown | molecular function unknown |
| YPL158C |  | biological process unknown | molecular function unknown |
| YPL208W | *RKM1* | biological process unknown | protein-lysine N-methyltransferase activity |
| YPL199C |  | biological process unknown | molecular function unknown |
| YPL263C | *KEL3* | biological process unknown | molecular function unknown |
| YMR144W |  | biological process unknown | molecular function unknown |
| YLR460C |  | biological process unknown | molecular function unknown |
| YCR016W |  | biological process unknown | molecular function unknown |
| YDR528W | *HLR1* | cell wall organization and biogenesis | molecular function unknown |
| YBR010W | *HHT1* | chromatin assembly or disassembly | DNA binding |
| YMR022W | *QRI8* | chromatin assembly or disassembly | ubiquitin-protein ligase activity |
| YAR018C | *KIN3* | chromosome segregation | protein kinase activity |
| YIR010W | *DSN1* | chromosome segregation | molecular function unknown |
| YPL209C | *IPL1* | chromosome segregation | protein kinase activity |
| YDL117W | *CYK3* | cytokinesis | molecular function unknown |
| YKL216W | *URA1* | 'de novo' pyrimidine base biosynthesis | dihydroorotate dehydrogenase activity |
| YAR007C | *RFA1* | DNA recombination | damaged DNA binding |
| YGL058W | *RAD6* | DNA repair | ubiquitin conjugating enzyme activity |
| YOR326W | *MYO2* | endocytosis | microfilament motor activity |
| YDR129C | *SAC6* | endocytosis | protein binding, bridging |
| YOR236W | *DFR1* | folic acid and derivative metabolism | dihydrofolate reductase activity |
| YJL187C | *SWE1* | G2/M transition of mitotic cell cycle | protein kinase activity |
| YGR108W | *CLB1* | G2/M transition of mitotic cell cycle | cyclin-dependent protein kinase regulator activity |
| YKL127W | *PGM1* | glucose 1-phosphate utilization | phosphoglucomutase activity |
| YGL084C | *GUP1* | glycerol transport | O-acyltransferase activity |
| YHR108W | *GGA2* | Golgi to vacuole transport | molecular function unknown |
| YDR484W | *VPS52* | Golgi to vacuole transport | protein binding |
| YDR027C | *VPS54* | Golgi to vacuole transport | molecular function unknown |
| YDR080W | *VPS41* | homotypic vacuole fusion, non- autophagic | Rab guanyl-nucleotide exchange factor activity |
| YMR319C | *FET4* | intracellular copper ion transport | iron ion transporter activity |
| YBR066C | *NRG2* | invasive growth (sensu Saccharomyces) | transcriptional repressor activity |
| YBR041W | *FAT1* | lipid transport | long-chain-fatty-acid-CoA ligase activity |
| YBL009W |  | meiosis | protein serine/threonine kinase activity |
| YPR141C | *KAR3* | meiosis | microtubule motor activity |
| YBR073W | *RDH54* | meiotic recombination | DNA-dependent ATPase activity |
| YLR180W | *SAM1* | methionine metabolism | methionine adenosyltransferase activity |
| YLR180W | *SAM1* | methionine metabolism | methionine adenosyltransferase activity |
| YPL124W | *SPC29* | microtubule nucleation | structural constituent of cytoskeleton |
| YML058W | *SML1* | mitochondrion organization and biogenesis | enzyme inhibitor activity |
| YOR249C | *APC5* | mitotic sister chromatid segregation | protein binding |
| YDR201W | *SPC19* | mitotic spindle organization and biogenesis in nucleus | structural constituent of cytoskeleton |
| YLR212C | *TUB4* | mitotic spindle organization and biogenesis in nucleus | structural constituent of cytoskeleton |
| YGL100W | *SEH1* | mRNA export from nucleus | structural molecule activity |
| YGL172W | *NUP49* | mRNA export from nucleus | structural molecule activity |
| YOL123W | *HRP1* | mRNA polyadenylylation | RNA binding |
| YMR299C | *DYN3* | nuclear migration, microtubule-mediated | motor activity |
| YER029C | *SMB1* | nuclear mRNA splicing, via spliceosome | RNA splicing factor activity, transesterification  mechanism |
| YOR241W | *MET7* | one-carbon compound metabolism | tetrahydrofolylpolyglutamate synthase activity |
| YNL130C | *CPT1* | phosphatidylcholine biosynthesis | diacylglycerol cholinephosphotransferase activity |
| YNL169C | *PSD1* | phosphatidylcholine biosynthesis | phosphatidylserine decarboxylase activity |
| YHR123W | *EPT1* | phosphatidylethanolamine biosynthesis | ethanolaminephosphotransferase activity |
| YER026C | *CHO1* | phosphatidylserine biosynthesis | CDP-diacylglycerol-serine  O-phosphatidyltransferase activity |
| YDR452W | *PPN1* | polyphosphate metabolism | endopolyphosphatase activity |
| YPL094C | *SEC62* | posttranslational protein targeting to membrane | protein binding |
| YBL056W | *PTC3* | protein amino acid dephosphorylation | protein phosphatase type 2C activity |
| YDL090C | *RAM1* | protein amino acid farnesylation | protein farnesyltransferase activity |
| YER027C | *GAL83* | protein amino acid phosphorylation | AMP-activated protein kinase activity |
| YKL048C | *ELM1* | protein amino acid phosphorylation | protein serine/threonine kinase activity |
| YJL006C | *CTK2* | protein amino acid phosphorylation | cyclin-dependent protein kinase regulator activity |
| YDL101C | *DUN1* | protein amino acid phosphorylation | protein kinase activity |
| YNL307C | *MCK1* | protein amino acid phosphorylation | glycogen synthase kinase 3 activity |
| YOR085W | *OST3* | protein complex assembly | dolichyl-diphosphooligosaccharide-protein  glycotransferase activity |
| YOR160W | *MTR10* | protein import into nucleus | nuclear localization sequence binding |
| YGL016W | *KAP122* | protein import into nucleus | protein carrier activity |
| YGL017W | *ATE1* | protein modification | arginyltransferase activity |
| YPL045W | *VPS16* | protein targeting to vacuole | molecular function unknown |
| YOR137C | *SIA1* | proton transport | molecular function unknown |
| YOR184W | *SER1* | purine base biosynthesis | phosphoserine transaminase activity |
| YOR101W | *RAS1* | Ras protein signal transduction | GTPase activity |
| YOR038C | *HIR2* | regulation of transcription from RNA polymerase II promoter | transcription corepressor activity |
| YMR179W | *SPT21* | regulation of transcription from RNA polymerase II promoter | molecular function unknown |
| YPL127C | *HHO1* | regulation of transcription, DNA-dependent | DNA binding |
| YNL021W | *HDA1* | regulation of transcription, DNA-dependent | histone deacetylase activity |
| YDR098C | *GRX3* | response to oxidative stress | thiol-disulfide exchange intermediate activity |
| YGR234W | *YHB1* | response to stress | nitric oxide reductase activity |
| YML014W | *TRM9* | response to stress | tRNA (uridine) methyltransferase activity |
| YHR012W | *VPS29* | retrograde transport, endosome to Golgi | molecular function unknown |
| YOL143C | *RIB4* | riboflavin biosynthesis | 6,7-dimethyl-8-ribityllumazine synthase activity |
| YBR265W | *TSC10* | sphingolipid biosynthesis | oxidoreductase activity, acting on NADH or  NADPH |
| YHR001W | *OSH7* | steroid biosynthesis | oxysterol binding |
| YPL069C | *BTS1* | terpenoid biosynthesis | farnesyltranstransferase activity |
| YML043C | *RRN11* | transcription from RNA polymerase I promoter | RNA polymerase I transcription factor activity |
| YOL145C | *CTR9* | transcription from RNA polymerase II promoter | RNA polymerase II transcription elongation factor activity |
| YNL257C | *SIP3* | transcription initiation from RNA polymerase II promoter | transcription cofactor activity |
| YER148W | *SPT15* | transcription initiation from RNA polymerase II promoter | DNA binding |
| YGR047C | *TFC4* | transcription initiation from RNA polymerase III promoter | RNA polymerase III transcription factor activity |
| YOR260W | *GCD1* | translational initiation | translation initiation factor activity |
| YOR259C | *RPT4* | ubiquitin-dependent protein catabolism | ATPase activity |
| YOL038W | *PRE6* | ubiquitin-dependent protein catabolism | endopeptidase activity |
| YPR108W | *RPN7* | ubiquitin-dependent protein catabolism | structural molecule activity |
| YCL063W | *VAC17* | vacuole inheritance | receptor activity |
| YPR029C | *APL4* | vesicle-mediated transport | clathrin binding |
| YPR028W | *YOP1* | vesicle-mediated transport | protein binding |
| YGR261C | *APL6* | vesicle-mediated transport | molecular function unknown |
| YPL195W | *APL5* | vesicle-mediated transport | protein binding |
| YER031C | *YPT31* | vesicle-mediated transport | GTPase activity |
