# Supplementary material for: Regulation of xylose metabolism in recombinant Saccharomyces cerevisiae
Source: Microb Cell Fact. 2008 Jun 4;7:18. doi: 10.1186/1475-2859-7-18 (PMC2435516; doi:10.1186/1475-2859-7-18)
Supplement: Additional file 9 — Cluster 4. List of open reading frames of in cluster 4 shown in Fig. 2 of the paper. [file 1475-2859-7-18-S9.doc]

### Additional file 9.

| **ORF** | Gene | **Process** | **Function** |
| --- | --- | --- | --- |
| YGR095C | *RRP46* | 35S primary transcript processing | 3'-5'-exoribonuclease activity |
| YKL051W | *SFK1* | actin cytoskeleton organization and  biogenesis | molecular function unknown |
| YOR355W | *GDS1* | aerobic respiration | molecular function unknown |
| YKR046C | *PET10* | aerobic respiration | molecular function unknown |
| YBR068C | *BAP2* | amino acid transport | amino acid transporter activity |
| YNL270C | *ALP1* | basic amino acid transport | basic amino acid transporter activity |
| YDL049C | *KNH1* | beta-1,6 glucan biosynthesis | molecular function unknown |
| YPL221W | *BOP1* | biological process unknown | molecular function unknown |
| YOR104W | *PIN2* | biological process unknown | molecular function unknown |
| YDR233C | *RTN1* | biological process unknown | molecular function unknown |
| YKR045C |  | biological process unknown | molecular function unknown |
| YDR242W | *AMD2* | biological process unknown | amidase activity |
| YOR233W | *KIN4* | biological process unknown | protein kinase activity |
| YPL176C | *TRE1* | biological process unknown | receptor activity |
| YHR078W |  | biological process unknown | molecular function unknown |
| YKL098W |  | biological process unknown | molecular function unknown |
| YJL082W | *IML2* | biological process unknown | molecular function unknown |
| YDR525W-A | *SNA2* | biological process unknown | molecular function unknown |
| YPR157W |  | biological process unknown | molecular function unknown |
| YKR075C |  | biological process unknown | molecular function unknown |
| YAL053W |  | biological process unknown | molecular function unknown |
| YCL049C |  | biological process unknown | molecular function unknown |
| YAR020C | *PAU7* | biological process unknown | molecular function unknown |
| YOR389W |  | biological process unknown | molecular function unknown |
| YNR073C |  | biological process unknown | mannitol dehydrogenase activity |
| YDR539W |  | biological process unknown | molecular function unknown |
| YBR085C-A |  | biological process unknown | molecular function unknown |
| YHL026C |  | biological process unknown | molecular function unknown |
| YDR441C | *APT2* | biological process unknown | molecular function unknown |
| YBL054W |  | biological process unknown | molecular function unknown |
| YLR375W | *STP3* | biological process unknown | molecular function unknown |
| YLR326W |  | biological process unknown | molecular function unknown |
| YKL071W |  | biological process unknown | molecular function unknown |
| YCR007C |  | biological process unknown | molecular function unknown |
| YKL151C |  | biological process unknown | molecular function unknown |
| YJL221C | *FSP2* | biological process unknown | alpha-glucosidase activity |
| YER037W | *PHM8* | biological process unknown | molecular function unknown |
| YEL041W |  | biological process unknown | molecular function unknown |
| YMR291W |  | biological process unknown | protein kinase activity |
| YOR338W |  | biological process unknown | molecular function unknown |
| YBR054W | *YRO2* | biological process unknown | molecular function unknown |
| YPL230W |  | biological process unknown | molecular function unknown |
| YPL222W | *FMP40* | biological process unknown | molecular function unknown |
| YLR202C |  | biological process unknown | molecular function unknown |
| YFR054C |  | biological process unknown | molecular function unknown |
| YMR103C |  | biological process unknown | molecular function unknown |
| YKR012C |  | biological process unknown | molecular function unknown |
| YAL060W | *BDH1* | butanediol fermentation | (R,R)-butanediol dehydrogenase activity |
| YGL248W | *PDE1* | cAMP-mediated signaling | cAMP-specific phosphodiesterase activity |
| SUC4 |  | carbohydrate metabolism | beta-fructofuranosidase activity |
| YGL156W | *AMS1* | carbohydrate metabolism | alpha-mannosidase activity |
| YGR023W | *MTL1* | cell wall organization and biogenesis | molecular function unknown |
| YDR055W | *PST1* | cell wall organization and biogenesis | molecular function unknown |
| YPL089C | *RLM1* | cell wall organization and biogenesis | DNA binding |
| YCR089W | *FIG2* | cellular morphogenesis during conjugation  with cellular fusion | molecular function unknown |
| YGL053W | *PRM8* | conjugation with cellular fusion | molecular function unknown |
| YML047C | *PRM6* | conjugation with cellular fusion | molecular function unknown |
| YER035W | *EDC2* | deadenylylation-dependent decapping | RNA binding |
| YIL066C | *RNR3* | DNA replication | ribonucleoside-diphosphate reductase activity |
| YHR142W | *CHS7* | ER to Golgi transport | molecular function unknown |
| YDR524C | *AGE1* | ER to Golgi transport | ARF GTPase activator activity |
| YIL107C | *PFK26* | fructose 2,6-bisphosphate metabolism | 6-phosphofructo-2-kinase activity |
| YGR256W | *GND2* | glucose metabolism | phosphogluconate dehydrogenase  (decarboxylating) activity |
| YFR053C | *HXK1* | glucose metabolism | hexokinase activity |
| YPL110C | *GDE1* | glycerophospholipid catabolism | glycerophosphocholine phosphodiesterase  activity |
| YOR036W | *PEP12* | Golgi to vacuole transport | t-SNARE activity |
| YJR158W | *HXT16* | hexose transport | glucose transporter activity |
| YPR193C | *HPA2* | histone acetylation | histone acetyltransferase activity |
| YBR295W | *PCA1* | iron ion homeostasis | ATPase activity, coupled to transmembrane  movement of ions, phosphorylative mechanism |
| YMR065W | *KAR5* | karyogamy during conjugation with cellular  fusion | molecular function unknown |
| YLR023C | *IZH3* | lipid metabolism | metal ion binding |
| YOL122C | *SMF1* | manganese ion transport | di-, tri-valent inorganic cation transporter activity |
| YPR007C | *REC8* | meiosis | binding |
| YDR517W | *GRH1* | mitotic spindle checkpoint | molecular function unknown |
| YBR093C | *PHO5* | phosphate metabolism | acid phosphatase activity |
| YML123C | *PHO84* | phosphate transport | manganese ion transporter activity |
| YPR005C | *HAL1* | positive regulation of transcription from  RNA polymerase II promoter | molecular function unknown |
| YOR119C | *RIO1* | processing of 20S pre-rRNA | protein kinase activity |
| YNL053W | *MSG5* | protein amino acid dephosphorylation | prenylated protein tyrosine phosphatase activity |
| YKR061W | *KTR2* | protein amino acid N-linked glycosylation | mannosyltransferase activity |
| YCR073C | *SSK22* | protein amino acid phosphorylation | MAP kinase kinase kinase activity |
| YOL113W | *SKM1* | protein amino acid phosphorylation | protein serine/threonine kinase activity |
| YDR247W | *VHS1* | protein amino acid phosphorylation | protein kinase activity |
| YOR003W | *YSP3* | protein catabolism | peptidase activity |
| YBR169C | *SSE2* | protein folding | molecular function unknown |
| YER154W | *OXA1* | protein import into mitochondrial inner membrane | protein transporter activity |
| YDR092W | *UBC13* | protein monoubiquitination | ubiquitin conjugating enzyme activity |
| YLR024C | *UBR2* | protein monoubiquitination | ubiquitin-protein ligase activity |
| YOR359W | *VTS1* | protein targeting to vacuole | RNA binding |
| YKR038C | *KAE1* | proteolysis | metalloendopeptidase activity |
| YMR016C | *SOK2* | pseudohyphal growth | transcription factor activity |
| YDL024C | *DIA3* | pseudohyphal growth | acid phosphatase activity |
| YMR322C | *SNO4* | pyridoxine metabolism | unfolded protein binding |
| YER064C |  | regulation of transcription | molecular function unknown |
| YBL088C | *TEL1* | response to DNA damage stimulus | protein kinase activity |
| YPR093C | *ASR1* | response to ethanol | molecular function unknown |
| YOR120W | *GCY1* | response to salt stress | aldo-keto reductase activity |
| YGR088W | *CTT1* | response to stress | catalase activity |
| YMR169C | *ALD3* | response to stress | aldehyde dehydrogenase activity |
| YDR214W | *AHA1* | response to stress | chaperone activator activity |
| YDR365C | *ESF1* | rRNA processing | RNA binding |
| YCR018C | *SRD1* | rRNA processing | molecular function unknown |
| YLR106C | *MDN1* | rRNA processing | ATPase activity |
| YGL121C | *GPG1* | signal transduction | signal transducer activity |
| YDR085C | *AFR1* | signal transduction during conjugation with cellular fusion | receptor signaling protein activity |
| YDL138W | *RGT2* | signal transduction | receptor activity |
| YDR277C | *MTH1* | signal transduction | molecular function unknown |
| YNL294C | *RIM21* | sporulation (sensu Fungi) | molecular function unknown |
| YNL202W | *SPS19* | sporulation (sensu Fungi) | 2,4-dienoyl-CoA reductase (NADPH) activity |
| YPL170W | *DAP1* | sterol metabolism | heme binding |
| YIL162W | *SUC2* | sucrose catabolism | beta-fructofuranosidase activity |
| YLL057C | *JLP1* | sulfur metabolism | sulfonate dioxygenase activity |
| YJR156C | *THI11* | thiamin biosynthesis | protein binding |
| YNL334C | *SNO2* | thiamin biosynthesis | molecular function unknown |
| YEL046C | *GLY1* | threonine catabolism | threonine aldolase activity |
| YNL314W | *DAL82* | transcription initiation from RNA polymerase II promoter | transcriptional activator activity |
| YOR306C | *MCH5* | transport | transporter activity |
| YDR384C | *ATO3* | transport | transporter activity |
| YPL058C | *PDR12* | transport | xenobiotic-transporting ATPase activity |
| YLR224W |  | ubiquitin-dependent protein catabolism | protein binding |
| YJL048C | *UBX6* | ubiquitin-dependent protein catabolism | molecular function unknown |
| YFL054C |  | water transport | transporter activity |
