# Supplementary material for: Regulation of xylose metabolism in recombinant Saccharomyces cerevisiae
Source: Microb Cell Fact. 2008 Jun 4;7:18. doi: 10.1186/1475-2859-7-18 (PMC2435516; doi:10.1186/1475-2859-7-18)
Supplement: Additional file 10 — Cluster 5. List of open reading frames in cluster 5 shown in Fig. 2 of the paper. [file 1475-2859-7-18-S10.doc]

### Additional file 10.

| **ORF** | Gene | **Process** | **Function** |
| --- | --- | --- | --- |
| YLL008W | *DRS1* | 35S primary transcript processing | ATP-dependent RNA helicase activity |
| YPL061W | *ALD6* | acetate biosynthesis | aldehyde dehydrogenase activity |
| YHL036W | *MUP3* | amino acid transport | L-methionine transporter activity |
| YBL029W |  | biological process unknown | molecular function unknown |
| YLL055W |  | biological process unknown | ion transporter activity |
| YOL164W | *BDS1* | dodecyl sulfate metabolism | arylsulfatase activity |
| YEL065W | *SIT1* | iron ion homeostasis | siderophore-iron (ferrioxamine) uptake  transporter activity |
| YNL277W | *MET2* | methionine biosynthesis | homoserine O-acetyltransferase activity |
| YDR502C | *SAM2* | methionine metabolism | methionine adenosyltransferase activity |
| YKL001C | *MET14* | methionine metabolism | adenylylsulfate kinase activity |
| YDR497C | *ITR1* | myo-inositol transport | myo-inositol transporter activity |
| YPR194C | *OPT2* | oligopeptide transport | oligopeptide transporter activity |
| YOR095C | *RKI1* | pentose-phosphate shunt | ribose-5-phosphate isomerase activity |
| YLL028W | *TPO1* | polyamine transport | spermine transporter activity |
| YER042W | *MXR1* | response to oxidative stress | protein-methionine-S-oxide reductase activity |
| YPL274W | *SAM3* | S-adenosylmethionine transport | S-adenosylmethionine transporter activity |
| YLL061W | *MMP1* | S-methylmethionine transport | S-methylmethionine transporter activity |
| YDR253C | *MET32* | sulfur amino acid metabolism | DNA binding |
| YJL212C | *OPT1* | sulfur metabolism | oligopeptide transporter activity |
| YIL119C | *RPI1* | thiamin biosynthesis | small GTPase regulator activity |
