# Supplementary material for: Regulation of xylose metabolism in recombinant Saccharomyces cerevisiae
Source: Microb Cell Fact. 2008 Jun 4;7:18. doi: 10.1186/1475-2859-7-18 (PMC2435516; doi:10.1186/1475-2859-7-18)
Supplement: Additional file 11 — Cluster 6. List of open reading frames of in cluster 6 shown in Fig. 2 of the paper. [file 1475-2859-7-18-S11.doc]

### Additional file 11.

| **ORF** | Gene | **Process** | **Function** |
| --- | --- | --- | --- |
| YMR081C | *ISF1* | aerobic respiration | molecular function unknown |
| YKL093W | *MBR1* | aerobic respiration | molecular function unknown |
| YBR298C | *MAL31* | alpha-glucoside transport | alpha-glucoside:hydrogen symporter activity |
| YGR289C | *MAL11* | alpha-glucoside transport | alpha-glucoside:hydrogen symporter activity |
| YHR033W |  | biological process unknown | molecular function unknown |
| YMR323W | *ERR3* | biological process unknown | phosphopyruvate hydratase activity |
| YMR118C |  | biological process unknown | molecular function unknown |
| YPL054W | *LEE1* | biological process unknown | molecular function unknown |
| YNL195C |  | biological process unknown | molecular function unknown |
| YKL107W |  | biological process unknown | molecular function unknown |
| YGR236C | *SPG1* | biological process unknown | molecular function unknown |
| YMR206W |  | biological process unknown | molecular function unknown |
| YIL136W | *OM45* | biological process unknown | molecular function unknown |
| YKL163W | *PIR3* | cell wall organization and biogenesis | structural constituent of cell wall |
| YPR030W | *CSR2* | cell wall organization and biogenesis | molecular function unknown |
| YDL223C | *HBT1* | cellular morphogenesis during conjugation  with cellular fusion | molecular function unknown |
| YFL030W | *AGX1* | glycine biosynthesis | alanine-glyoxylate transaminase activity |
| YBR299W | *MAL32* | maltose catabolism | alpha-glucosidase activity |
| YJL116C | *NCA3* | mitochondrion organization and biogenesis | molecular function unknown |
| YBR117C | *TKL2* | pentose-phosphate shunt | transketolase activity |
| YPL223C | *GRE1* | response to stress | molecular function unknown |
| YNR002C | *ATO2* | transport | transporter activity |
