# Supplementary material for: Regulation of xylose metabolism in recombinant Saccharomyces cerevisiae
Source: Microb Cell Fact. 2008 Jun 4;7:18. doi: 10.1186/1475-2859-7-18 (PMC2435516; doi:10.1186/1475-2859-7-18)
Supplement: Additional file 12 — Cluster 7. List of open reading frames in cluster 7 shown in Fig. 2 of the paper. [file 1475-2859-7-18-S12.doc]

### Additional file 12.

| **ORF** | Gene | **Process** | **Function** |
| --- | --- | --- | --- |
| YLR194C |  | biological process unknown | molecular function unknown |
| YLR042C |  | biological process unknown | molecular function unknown |
| YBR137W |  | biological process unknown | molecular function unknown |
| YMR315W |  | biological process unknown | molecular function unknown |
| YDR222W |  | biological process unknown | molecular function unknown |
| YCR045C |  | biological process unknown | serine-type peptidase activity |
| YLR073C |  | biological process unknown | molecular function unknown |
| YGR039W |  | biological process unknown | molecular function unknown |
| YMR108W | *ILV2* | branched chain family amino acid  biosynthesis | acetolactate synthase activity |
| YHR208W | *BAT1* | branched chain family amino acid  biosynthesis | branched-chain-amino-acid transaminase activity |
| YGR049W | *SCM4* | cell cycle | molecular function unknown |
| YJL108C | *PRM10* | conjugation with cellular fusion | molecular function unknown |
| YIL117C | *PRM5* | conjugation with cellular fusion | molecular function unknown |
| YER073W | *ALD5* | electron transport | aldehyde dehydrogenase activity |
| YGR166W | *KRE11* | ER to Golgi transport | molecular function unknown |
| YOL136C | *PFK27* | fructose 2,6-bisphosphate metabolism | 6-phosphofructo-2-kinase activity |
| YHR092C | *HXT4* | hexose transport | glucose transporter activity |
| YHR046C | *INM1* | inositol phosphate dephosphorylation | inositol-1(or 4)-monophosphatase activity |
| YOR226C | *ISU2* | iron ion homeostasis | molecular function unknown |
| YPL192C | *PRM3* | karyogamy | molecular function unknown |
| YGL009C | *LEU1* | leucine biosynthesis | 3-isopropylmalate dehydratase activity |
| YCL018W | *LEU2* | leucine biosynthesis | 3-isopropylmalate dehydrogenase activity |
| YOR108W | *LEU9* | leucine biosynthesis | 2-isopropylmalate synthase activity |
| YMR316W | *DIA1* | pseudohyphal growth | molecular function unknown |
| YLR044C | *PDC1* | pyruvate metabolism | pyruvate decarboxylase activity |
| YGL089C | *MF(ALPHA)2* | response to pheromone during conjugation  with cellular fusion | mating pheromone activity |
| YDR211W | *GCD6* | translational initiation | translation initiation factor activity |
| YLR401C | *DUS3* | tRNA modification | tRNA dihydrouridine synthase activity |
