# Supplementary material for: Regulation of xylose metabolism in recombinant Saccharomyces cerevisiae
Source: Microb Cell Fact. 2008 Jun 4;7:18. doi: 10.1186/1475-2859-7-18 (PMC2435516; doi:10.1186/1475-2859-7-18)
Supplement: Additional file 13 — Cluster 8. List of open reading frames in cluster 8 shown in Fig. 2 of the paper. [file 1475-2859-7-18-S13.doc]

### Additional file 13.

| **ORF** | **Gene** | **Process** | **Function** |
| --- | --- | --- | --- |
| YNL047C | *SLM2* | actin cytoskeleton organization and  biogenesis | phosphoinositide binding |
| YOL111C |  | biological process unknown | molecular function unknown |
| YNL295W |  | biological process unknown | molecular function unknown |
| YMR160W |  | biological process unknown | molecular function unknown |
| YKR105C |  | biological process unknown | molecular function unknown |
| YGR021W |  | biological process unknown | molecular function unknown |
| YFL034W |  | biological process unknown | molecular function unknown |
| YDR185C |  | biological process unknown | molecular function unknown |
| YMR195W | *ICY1* | biological process unknown | molecular function unknown |
| YKR051W |  | biological process unknown | molecular function unknown |
| YJL217W |  | biological process unknown | molecular function unknown |
| YGL010W |  | biological process unknown | molecular function unknown |
| YDL133W |  | biological process unknown | molecular function unknown |
| YGR079W |  | biological process unknown | molecular function unknown |
| YCL041C |  | biological process unknown | molecular function unknown |
| YJR160C | *MPH3* | carbohydrate transport | carbohydrate transporter activity |
| YLR425W | *TUS1* | cell wall organization and biogenesis | Rho guanyl-nucleotide exchange factor activity |
| YLR357W | *RSC2* | chromatin remodeling | molecular function unknown |
| YPL022W | *RAD1* | DNA recombination | single-stranded DNA specific  endodeoxyribonuclease activity |
| YMR137C | *PSO2* | DNA repair | damaged DNA binding |
| YBR170C | *NPL4* | ER-associated protein catabolism | molecular function unknown |
| YOR188W | *MSB1* | establishment of cell polarity (sensu Fungi) | molecular function unknown |
| YPL174C | *NIP100* | establishment of mitotic spindle orientation | protein binding |
| YPL113C |  | metabolism | oxidoreductase activity |
| YBL063W | *KIP1* | microtubule nucleation | structural constituent of cytoskeleton |
| YFL036W | *RPO41* | mitochondrial genome maintenance | DNA-directed RNA polymerase activity |
| YLR304C | *ACO1* | mitochondrial genome maintenance | aconitate hydratase activity |
| YIL051C | *MMF1* | mitochondrial genome maintenance | molecular function unknown |
| YOR211C | *MGM1* | mitochondrion organization and biogenesis | GTPase activity |
| YGL216W | *KIP3* | mitotic spindle organization and biogenesis  in nucleus | microtubule motor activity |
| YJR050W | *ISY1* | nuclear mRNA splicing, via spliceosome | RNA splicing factor activity, transesterification  mechanism |
| YMR204C | *INP1* | peroxisome inheritance | molecular function unknown |
| YDR174W | *HMO1* | plasmid maintenance | single-stranded DNA binding |
| YBR057C | *MUM2* | premeiotic DNA synthesis | molecular function unknown |
| YDR462W | *MRPL28* | protein biosynthesis | structural constituent of ribosome |
| YBR037C | *SCO1* | protein complex assembly | thioredoxin peroxidase activity |
| YDL113C | *ATG20* | protein targeting to vacuole | lipid binding |
| YER078C |  | proteolysis | metalloendopeptidase activity |
| YKR052C | *MRS4* | RNA splicing | iron ion transporter activity |
| YAL056W | *GPB2* | signal transduction | signal transducer activity |
| YMR240C | *CUS1* | spliceosome assembly | protein binding |
| YBR045C | *GIP1* | spore wall assembly (sensu Fungi) | protein phosphatase 1 binding |
| YDL005C | *MED2* | transcription from RNA polymerase II promoter | RNA polymerase II transcription mediator activity |
| YKR089C | *TGL4* | triacylglycerol mobilization | triacylglycerol lipase activity |
| YOR136W | *IDH2* | tricarboxylic acid cycle | isocitrate dehydrogenase (NAD+) activity |
| YGR003W | *CUL3* | ubiquitin-dependent protein catabolism | protein binding |
| YML088W | *UFO1* | ubiquitin-dependent protein catabolism | protein binding |
| YJR104C | *SOD1* | zinc ion homeostasis | copper, zinc superoxide dismutase activity |
