# Supplementary material for: Regulation of xylose metabolism in recombinant Saccharomyces cerevisiae
Source: Microb Cell Fact. 2008 Jun 4;7:18. doi: 10.1186/1475-2859-7-18 (PMC2435516; doi:10.1186/1475-2859-7-18)

**Additional file 14.** Image of the 11% SDS-PAGE 2-DE-gel showing the locations of the seventy protein spots with different abundance in cells growing on glucose or xylose. (pI range 3-10 from left to right)


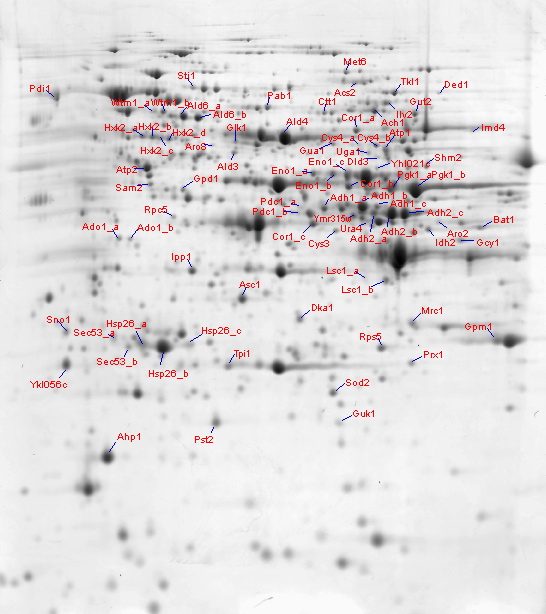

Supplement: Additional file 14 — Image of the 11% SDS-PAGE 2-DE-gel. The image of the 2-DE-gel showing the locations of the seventy protein spots, which had different abundance in cells growing on glucose or xylose. [file 1475-2859-7-18-S14.doc]
