# Supplementary material for: Regulation of xylose metabolism in recombinant Saccharomyces cerevisiae
Source: Microb Cell Fact. 2008 Jun 4;7:18. doi: 10.1186/1475-2859-7-18 (PMC2435516; doi:10.1186/1475-2859-7-18)

**Additional file 15.** Seventy proteins which were differentially translated in the glucose repressed, glucose derepressed and xylose-grown cells, identified from 2-DE gels. Proteins are clustered by using hierarchical clustering with Euclidean distance and average linkage. The x-axis corresponds to the samples taken at 5 h and 24 h from glucose (Glc) cultures and at 72 h from xylose (Xyl) cultures.


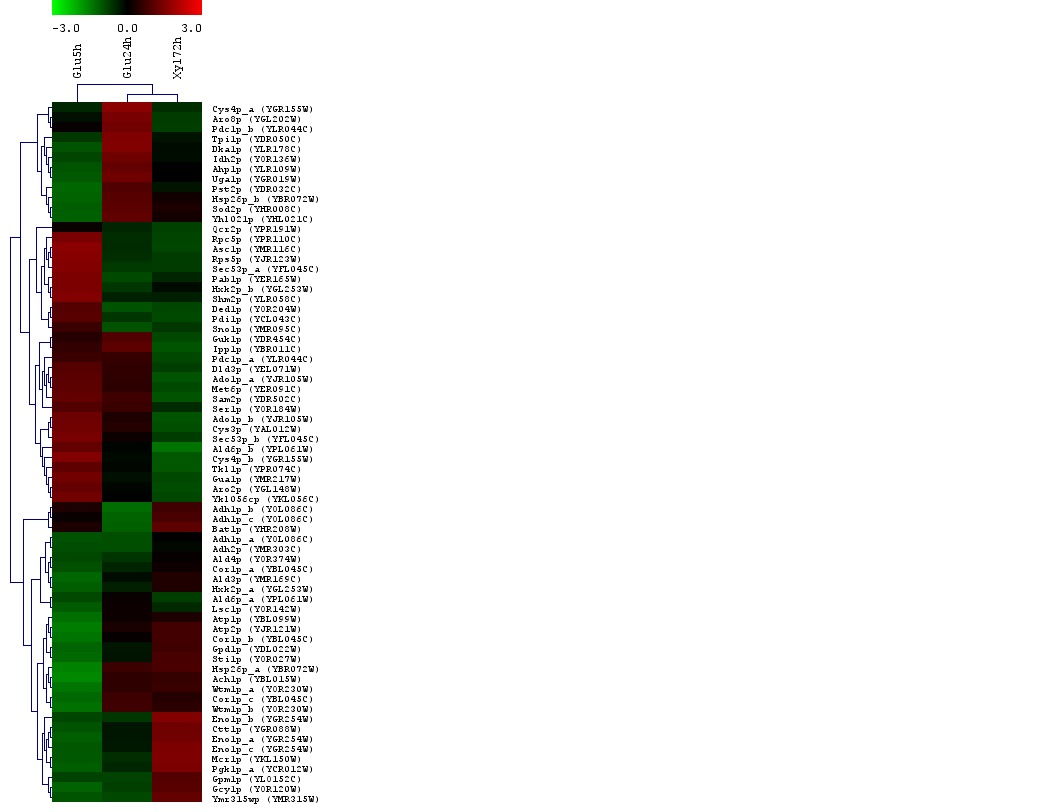

Supplement: Additional file 15 — Clustering of the proteome data. The figure shows seventy proteins, which were differentially translated in the glucose repressed, glucose derepressed and xylose-grown cells and clustered by using hierarchical clustering with Euclidean distance and average linkage. [file 1475-2859-7-18-S15.doc]
