# Supplementary material for: Regulation of xylose metabolism in recombinant Saccharomyces cerevisiae
Source: Microb Cell Fact. 2008 Jun 4;7:18. doi: 10.1186/1475-2859-7-18 (PMC2435516; doi:10.1186/1475-2859-7-18)
Supplement: Additional file 16 — Expression profiles of genes involved in cyclic AMP – phosphokinase A pathway (cAMP-PKA) of S. cerevisiae. The figure provided shows the expression trend of genes involved in cyclic AMP – phosphokinase A pathway in cells grown on glucose for 5 or 24 h or on xylose for 72 h. [file 1475-2859-7-18-S16.doc]

**Additional file 16.** Expression profiles of genes involved in cyclic AMP – phosphokinase A pathway (cAMP-PKA) of *S. cerevisiae*. Transcription factors are presented with boldfacing and connected to respective target genes with gray lines. Expression of genes presented in white boxes with black text was highest in derepressed cells (Derep.) and lowest in glucose repressed cells (Rep.). Expression of genes presented in black boxes with white text was highest in glucose repressed cells and lowest in derepressed cells. Expression of genes presented in dark gray boxes with black text was highest in xylose-grown cells and lowest in derepressed cells. Expression of genes presented in light gray boxes with black text was highest in xylose-grown cells and lowest in glucose repressed cells. Expression of genes presented in gray boxes with white text was lowest in xylose-grown cells.
