# Supplementary material for: Regulation of xylose metabolism in recombinant Saccharomyces cerevisiae
Source: Microb Cell Fact. 2008 Jun 4;7:18. doi: 10.1186/1475-2859-7-18 (PMC2435516; doi:10.1186/1475-2859-7-18)
Supplement: Additional file 17 — Images of 2-DE gels with phosphorylated proteins. Images of 2-DE gels showing the locations of Hxk2p, Glk1p, Eno2p and Eno1p in samples from cells grown for 72 h on xylose and for 5 h on glucose and stained either with phosphoprotein specific Pro-Q Diamond or Sypro Ruby. [file 1475-2859-7-18-S17.doc]

**Additional file 17.** Images of 2-DE gels showing the locations of hexokinase 2 (Hxk2), glucokinase (Glk1), enolase 2 (Eno2), and enolase 1 (Eno1) in samples from cells grown for 72 h on xylose and for 5 h on glucose and stained either with phosphoprotein specific Pro-Q Diamond (upper) or Sypro Ruby (lower).
